# Robust bearing fault diagnosis for rotating machinery under noisy and variable operating conditions using a Wavelet-Mamba Network

**DOI:** 10.1371/journal.pone.0358759

**Published:** 2026-09-28

**Authors:** Dayou Cui, Zhaoyan Xie, Zhixue Wang, Xiaowei Li, Xiangxuan Meng

**Affiliations:** School of Railway Transportation, Shandong Jiaotong University, Jinan, China; Xi’an Jiaotong University, CHINA

## Abstract

Bearing fault diagnosis for rotating machinery remains challenging due to strong background noise and variable operating conditions in harsh industrial environments. To address these issues, this paper proposes a robust and lightweight bearing fault diagnosis framework, termed Multi-scale and Dual-scale Wavelet Mamba (MDW-Mamba), for noisy and non-stationary industrial applications. The proposed method employs a multi-stage “noise-feature decoupling” strategy, comprising a statistically guided three-channel input, a Multi-scale Omni-kernel Convolutions (MOM-Conv) backbone, and a Dual-Scale Wavelet Mamba (DSW-Mamba) module. This architecture explicitly separates fault components from broadband noise while enabling efficient long-term dependency modeling. Experimental results on three public datasets under variable-speed conditions demonstrate that MDW-Mamba consistently outperforms state-of-the-art methods. It achieves an average classification accuracy exceeding 87% even at a signal-to-noise ratio of −6 dB, showing exceptional robustness against strong interference. Furthermore, the model maintains an ultra-lightweight footprint of 0.26 M parameters and a fast inference time of 1.285 ms, indicating strong potential for real-time, on-board deployment in resource-constrained industrial edge devices.

## Introduction

The efficient operation of modern industrial sectors relies heavily on the reliability of critical rotating assets, such as wind turbines, aerospace propulsion systems, and heavy-duty manufacturing equipment [1,2]. As critical “joints” of this equipment, the health status of rolling bearings directly determines the safety and efficiency of complex industrial processes [3]. Unlike stationary equipment in controlled laboratory environments, critical rotating machinery often operates for extended periods in harsh and unpredictable industrial settings. These machines face extremely challenging conditions, enduring violent vibrations, variable alternating impact loads, and complex environmental interference [4]. Statistics indicate that approximately 45%−55% of rotating machinery failures originate from bearing damage [5]. Consequently, failures during critical production phases result in severe downtime losses and catastrophic economic consequences.

A major challenge is that rotational speeds of industrial equipment fluctuate in real-time according to varying load demands and complex duty cycles. The resulting non-stationary signals are easily submerged by strong background noise, making weak fault features difficult to extract [6]. Recently, with the advancement of Industrial 4.0 and Intelligent Manufacturing, Deep Learning (DL) methods have gradually overcome the limitations of traditional signal processing. Due to their adaptive feature extraction capabilities, they have become the mainstream research direction for fault diagnosis industrial rotating machinery [7].

Although deep learning has advanced mechanical fault diagnosis, existing algorithms face challenges under the intense time-varying and high-noise conditions common in complex industrial equipment. Classic CNNs, such as WDCNN [8] and ResNet18 [9], are widely used for feature extraction, while Multi-Scale CNNs (MSCNN [10]) capture multi-granularity fault patterns. However, their local receptive fields limit the capture of global temporal dependencies in long-sequence signals, making them prone to misdiagnosis under strong interference [11]. To fuse spatiotemporal features, hybrid models like CNN-LSTM [12] and DCA-BiGRU [13] utilize recurrent units; however, their serial computation leads to low efficiency and gradient vanishing issues. More recently, domain adaptation and incremental learning strategies have been introduced to handle cross-domain diagnosis under varying operating conditions [14,15], yet these approaches still rely on traditional backbone architectures without addressing the underlying noise-feature mixing problem. Recently, Transformers (e.g., TAR-1D [16]) have achieved global modeling via self-attention, but their quadratic complexity *O*(*L*^2^) imposes a heavy burden on resource-constrained industrial edge-side monitoring devices [17]. In the end, most of the current methods lack a clear frequency domain selection mechanism. In complex broadband noise environments, they often fail to effectively distinguish fault characteristics from random interference, resulting in a significant decrease in diagnostic accuracy.

To solve the previously mentioned problems of computational bottlenecks and limited sensing fields, structured state space models (SSMs) represented by mamba have emerged in recent years, which have received great attention as a new sequence model [18,19]. Benefiting from its innovative Selective Scan Mechanism, Mamba not only possesses global long-range dependency capturing capabilities comparable to Transformers but also achieves linear computational complexity *O*(*L*) that grows with sequence length. This characteristic has shown great potential in fields such as computer vision [20] and long-sequence time series forecasting [21], providing an ideal infrastructure for the lightweight deployment of intelligent diagnostic systems for rotating machinery.

Although the standard Mamba model has high computing efficiency, it still has inherent limitations when directly applied to fault diagnosis in noisy and complex industrial environments. Recent studies [22,23] show that the pure time domain architecture often fails to make full use of the sparseness of the frequency domain of mechanical vibration signals. As a state space sequence model, Mamba lacks a clear mechanism dedicated to frequency selection and decoupling. Therefore, in the broadband Gaussian noise environment omnipresent in modern industrial operations, it is difficult for the model to distinguish between high-frequency noise components and transient fault pulses, which leads to a serious “feature-noise” mixing phenomenon.

To enhance feature extraction from non-stationary signals, the Discrete Wavelet Transform (DWT) serves as a robust multi-resolution analysis tool [24]. By decomposing signals into sparse sub-bands, DWT facilitates the effective separation of noise from informative components. Therefore, integrating DWT-derived frequency-domain sparsity with Mamba’s global temporal modeling provides a promising framework for developing noise-resistant yet inference-efficient diagnostic architectures for various rotating machinery, such as the proposed MDW-Mamba model.

Recent cross-domain research has begun exploring frequency-domain perception. In computer vision, WaveMamba [25] and IRSRMamba [26] integrated wavelet transforms with Mamba to enhance texture and structural modeling in 2D image tasks. Following this, Yang et al. [27] applied Discrete Multi-Wavelet Transform (DMWT) with Mamba to address few-shot fault diagnosis, where the primary challenge is data scarcity rather than noise interference. More recently, Lai et al. [28] combined CNN and Mamba-enhanced LSTM for bearing fault diagnosis, yet their approach focuses on multi-channel feature fusion without explicit frequency-domain noise decoupling.

Despite these advances, existing wavelet-Mamba methods differ fundamentally from our work in two aspects. First, prior 1D fault diagnosis works [27,28] employ wavelets primarily as a feature enrichment tool under limited data conditions, rather than as a noise-feature decoupling mechanism under extreme noise. Second, they have not explored wavelet-guided selective state scanning for 1D strong background noise separation, nor designed specific frequency-drift compensation mechanisms for non-stationary industrial operating conditions where rotational speed varies continuously. The proposed MDW-Mamba addresses these gaps by integrating DWT as a frequency-selective prior that explicitly guides Mamba’s state update process to prioritize fault-related subbands while suppressing broadband noise, achieving robust diagnosis at SNR as low as −6 dB.

Addressing these challenges, this paper proposes the MDW-Mamba network with the “noise-feature decoupling” mechanism as the core. Based on the research results of Lai et al. [28], the framework adopts a three-channel input strategy based on statistical filtering and combines with the mixed multi-view convolution (MOM-Conv) backbone network to capture local multi-scale features and achieve initial noise reduction. The core innovation point solves Mamba’s limitations in frequency domain selectivity on the basis of the framework proposed by Yang et al. [27].Unlike the previous “wavelet + Mamba” method, which mainly aimed at the complement of fewer sample features, this study uses the inherent frequency sparseness of wavelet transformation to guide the selective state scanning mechanism of Mamba. By focusing on a specific subband dominated by fault-induced transients, the model can achieve effective physical separation of fault-related characteristics and noise even under extreme conditions of −6 decibels.

The main contributions of this paper are as follows:

(1) The MDW-Mamba framework is proposed for the fault diagnosis of rotating machinery in high-noise industrial environments. By combining statistical filtering with MOM-Conv, the framework overcomes the problems of limited sensing field of convolutional neural networks (CNNs) and high computational complexity of Transformer, and achieves an effective balance between diagnostic accuracy and reasoning efficiency.
(2) The DSW-Mamba module has been developed to introduce frequency domain perception capabilities. Unlike prior wavelet-Mamba approaches that use wavelets primarily for feature enrichment under data scarcity [27] or vision tasks [25], our module employs discrete wavelet transformation (DWT) to explicitly realize “noise-feature decoupling” and guides Mamba’s selective state update mechanism to focus exclusively on the subband containing fault-related information. This design addresses the spectral mixing problem under broadband noise, significantly improving the robustness of the model under extreme noise conditions.
(3) A large number of experiments conducted on three public data sets prove the superiority of MDW-Mamba. It has only 260,000 parameters, which is superior to the mainstream model in noisy environments and non-stationary operating conditions, which confirms its potential for real-time and edge-side deployment in industrial condition monitoring.

## Proposed method

### MDW-Mamba overview

In order to achieve high-precision fault diagnosis of rotating machinery under strong noise and complex industrial environments, this paper proposes a hierarchical architecture called MDW-Mamba (Multi-scale and Dual-scale Wavelet Mamba). As shown in Fig 1, this architecture is specially designed to cope with challenges such as speed fluctuations, load changes and broadband environmental noise common in modern industrial processes. It overcomes the limited sensing field of traditional convolutional neural networks (CNNs) [29] and the problem that Transformer calculates too much complexity when modeling long sequences [30]. These problems often hinder their actual deployment on resource-constrained industrial edge devices.

**Fig 1:**
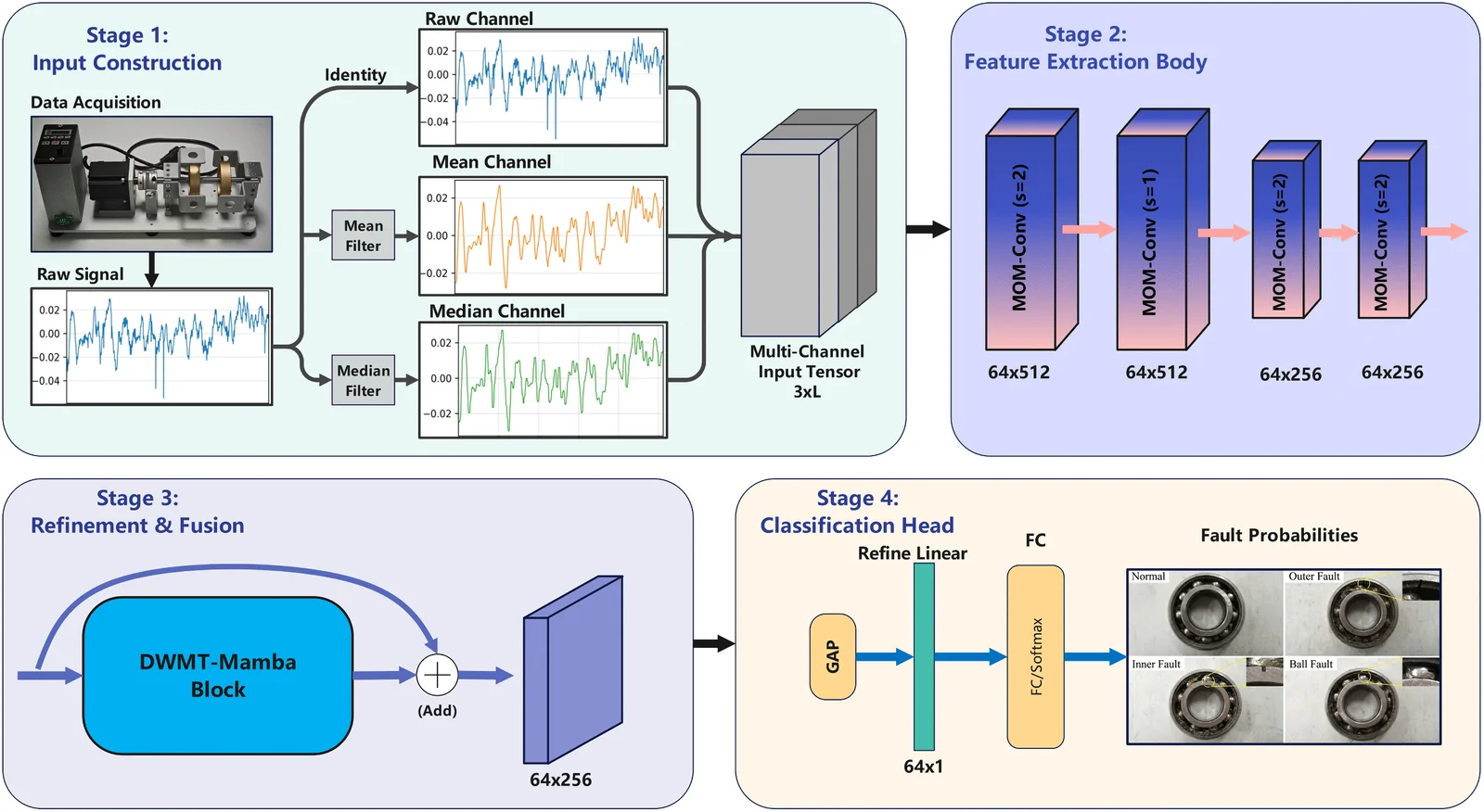
The overall architecture of the proposed MDW-Mamba framework. The hierarchical architecture for bearing fault diagnosis comprises four sequential stages: multi-channel input construction, a multi-scale feature extraction backbone (MOM-Conv), dual-scale refinement and fusion (DSW-Mamba), and a classification head.

The workflow of MDW-Mamba includes three consecutive stages: multi-channel signal reconstruction, adaptive multi-scale feature extraction and global time-frequency refinement. First of all, the model does not directly process the original one-dimensional signal, but uses the prior knowledge of statistical filtering to reconstruct the single-variable sequence into a three-channel tensor with physical significance to improve the signal-to-noise ratio of the input signal. Then, the data enters the backbone network composed of stacked multi-scale Omni-Kernel Convolution (MOM-Conv) modules. Unlike fixed nuclear convolution, MOM-Conv integrates the characteristics of different expansion rate branches through the adaptive gate control mechanism, and can capture short-term transient signals and long-term periodic fluctuations at the same time. In order to further suppress noise and capture global dependencies, the Dual-Scale Wavelet Mamba (DSW-Mamba) module adopts Discrete Wavelet Transform, (DWT) decouples the input signal into low-frequency and high-frequency subbands. This module uses Mamba to model long program columns of low-frequency components, and filters out high-frequency noise with the help of the convolutional layer. Finally, local and global characteristics are integrated through residual connections, and the failure probability is generated through the Global Average Pooling (GAP) layer and the subsequent full connection layer.

### Multi-channel data construction

Existing single-channel data-driven methods often exhibit limited robustness under non-stationary operating conditions and strong background noise. To address this limitation, we construct a three-channel input tensor $𝒳\in {\mathbb{R}}^{3\times L}$, where *L* denotes the signal sampling length. The first channel, ${𝐱}_{raw}$, preserves the original time-domain waveform without distortion to prevent information loss. The second channel, ${𝐱}_{mean}$, is generated using a moving average filter with a sliding window of size k, designed to suppress zero-mean Gaussian white noise and smooth signal trends. The calculation is defined as:


$$\begin{array}{c} x_{mean,t}=\frac{1}{k}\sum_{j=-\lfloor k/2\rfloor }^{\lfloor k/2\rfloor }s_{t+j} \end{array}$$ (1)

The third channel, ${𝐱}_{med}$, employs non-linear median filtering, a technique that effectively suppresses impulsive noise and outliers commonly encountered in rotating machinery environments while preserving critical signal edge features, such as fault impact boundaries. Its mathematical expression is:


$$\begin{array}{c} x_{med,t}=median(\{s_{t-\lfloor k/2\rfloor },...,s_{t+\lfloor k/2\rfloor }\}) \end{array}$$ (2)

Finally, by concatenating these components along the channel dimension, the input tensor is constructed as:


$$\begin{array}{c} {𝒳}_{t}=[x_{raw,t},x_{mean,t},x_{med,t}{]}^{T} \end{array}$$ (3)

This mechanism enables the model to synchronously identify and learn fault patterns from three different signal dimensions, which effectively improves the robustness of the model against noise interference.

### Multi-Scale Omni-Kernel Convolution (MOM-Conv) module

The waveform of the bearing fault signal usually presents complex multi-scale characteristics: the accurate positioning of the weak early fault characteristics needs to be completed by relying on a small receptive field; while the capture of the periodic characteristics of the complex modulation edge frequency band requires a larger receptive field. The traditional convolutional neural network (CNN) architecture adopts a fixed convolutional kernel size, which is difficult to reconcile this inherent contradiction. In response to this problem, this study proposes a MOM-Conv module based on the idea of full-dimensional dynamic convolution. Its structure is shown in Fig 2.

**Fig 2:**
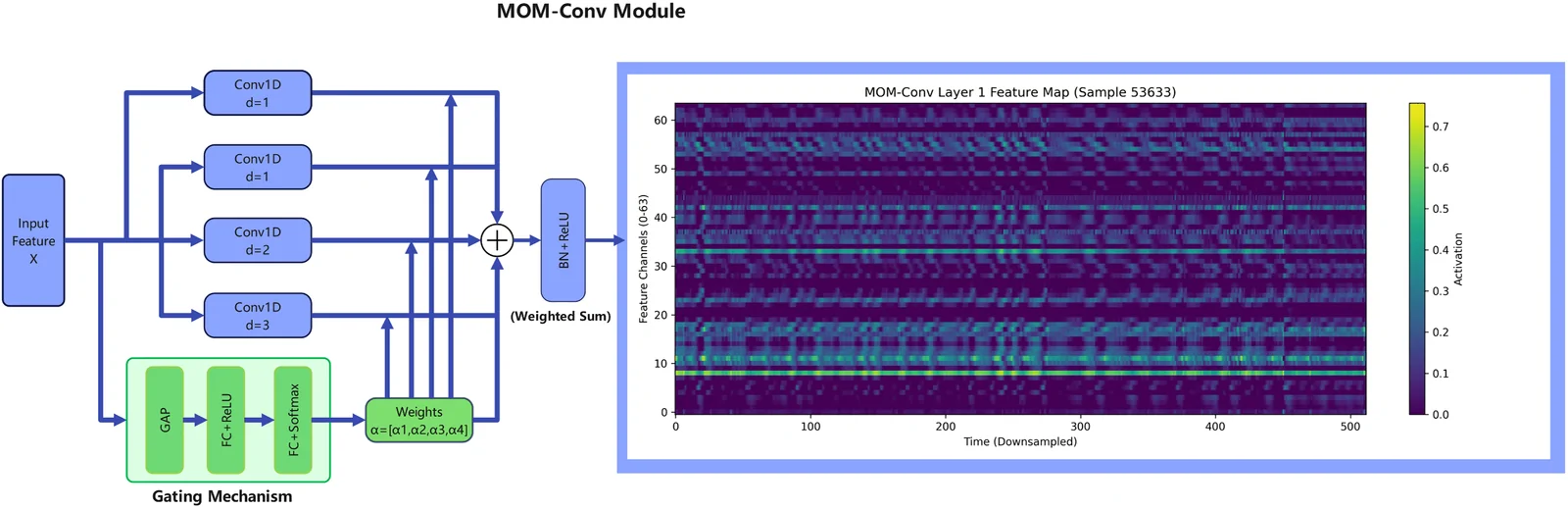
Architecture and feature visualization of the Multi-Scale Omni-Kernel Convolution (MOM-Conv) module. The left subplot presents the multi-branch dilated convolution structure integrated with an adaptive gating mechanism. The right subplot provides a representative visualization of the feature maps derived from the module’s output on the University of Ottawa dataset, which verifies the module’s capability to effectively extract periodic fault-related features while suppressing background noise components.

The module adopts a multi-branch architecture, which enables the model to simultaneously capture local fault transient characteristics and global periodic modulation characteristics by introducing parallel dilated low convolution with different expansion rates [31]. This multi-resolution feature extraction ability is crucial to ensure the robustness of fault diagnosis under harsh industrial operating conditions. As shown in the upper half of Fig 2, the structure of the module contains 4 parallel convolution branches; in order to capture the fault impact characteristics of different scales, each branch adopts the same convolutional kernel size and only configures different expansion rates. Considering that the early fault signal of the bearing is often manifested as an extremely weak short-term shock pulse, this study designed two branches with an expansion rate of 1 to strengthen the ability to capture high-frequency local details. This strategy effectively takes into account the precise positioning of transient shock characteristics and the context modeling needs of periodic modulation edge frequency bands. Let the input feature map be ${𝒳}_{input}$. The output of the *i*-th branch, ${\tilde{𝐘}}_{i}$, represents the feature representation at different temporal resolutions. To intelligently fuse these multi-scale features, we employ a Gating Network [32,33]. First, Global Average Pooling (GAP) is employed to extract the global context descriptor. Subsequently, channel attention weights $\alpha =[\alpha_{1},...,\alpha_{4}]$ are generated through two fully connected layers (parameterized by weight matrices ${𝐖}_{1}$ and ${𝐖}_{2}$) followed by a Softmax function, calculated as:


$$\begin{array}{c} \alpha =Softmax({𝐖}_{2}\delta ({𝐖}_{1}GAP({𝒳}_{input}))) \end{array}$$ (4)

where δ represents the Rectified Linear Unit (ReLU) activation function. The final output feature $F_{MOM}$ is derived by weighted fusion of each branch output, with subsequent Batch Normalization (BN) and nonlinear activation:


$$\begin{array}{c} F_{MOM}=ReLU\left(BN\left(\sum_{i=1}^{4}\alpha_{i}\cdot {\tilde{𝐘}}_{i}\right)\right) \end{array}$$ (5)

The omni-kernel design enables the network to dynamically adjust the effective receptive field according to the specific frequency characteristics of the input signal, which significantly improves the flexibility and accuracy of feature extraction.

For visual validation of the multi-scale structure design, the feature map visualization output of the first MOM-Conv layer is given on the right side of Fig 2. It can be seen that the vertical stripes with obvious periodicity in the feature diagram correspond to the repeated fault shock signal, and the dark background reflects the effective suppression of irrelevant noise. This result shows that the designed multi-dilated parallel branch can accurately capture the instantaneous impact characteristics and periodic modulation characteristics of the signal.

### Dual-Scale Wavelet Mamba Transform (DSW-Mamba)

Convolutional neural networks (CNNs) are good at local feature extraction, but the limitations of their receptive fields make it difficult to effectively model the long-range dependent features of fault evolution. Although Transformer has excellent global modeling capabilities, it is limited by the complexity of quadratic computing, and it is difficult to adapt to long-sequence signal analysis tasks. The Mamba architecture based on the selective state space model (SSMs), although the computational complexity is reduced to the linear level, it is directly used for noisy high-frequency vibration signals, which is very prone to overfitting.

The inherent suitability of SSMs for low-frequency modeling stems from their mathematical formulation. From a state-space perspective, the continuous-time dynamics 𝐀 in Eq 7 govern the rate at which past information is propagated or attenuated. Low-frequency signals, characterized by smooth temporal evolution and strong inter-sample correlations, align naturally with the Markovian structure of the hidden state *h*(*t*), where the state upda*t*e $\dot{h}(t)=𝐀h(t)+𝐁u(t)$ acts as an implicit low-pass filter by exponentially decaying high-frequency perturbations relative to the system’s eigendynamics. Furthermore, during discretization via Zero-Order Hold (ZOH), the timescale parameter Δ introduces a Nyquist-type constraint: frequencies exceeding 1/(2Δ) are inherently aliased and cannot be reliably encoded [18]. This means that when raw high-frequency vibration signals containing broadband noise are directly fed into the Mamba encoder, the stochastic fluctuations in the noise-dominant bands force the state transition matrices to fit spurious high-frequency patterns, leading to overfitting. Conversely, the low-frequency approximation coefficients ${𝐗}_{L}$ produced by DWT possess a compact spectral distribution that matches the SSM’s implicit bandwidth constraints, enabling stable and robust long-range dependency modeling without overfitting.

In response to the inherent short board of these three types of networks, this paper designs a dual-scale wavelet Mamba (DSW-Mamba) module (structure shown in Fig 3). Adopting a “divide-and-conquer” strategy, this module leverages the orthogonality of the Discrete Wavelet Transform (DWT) to construct a dual-scale feature space, aiming to balance modeling efficiency with noise robustness.

**Fig 3:**
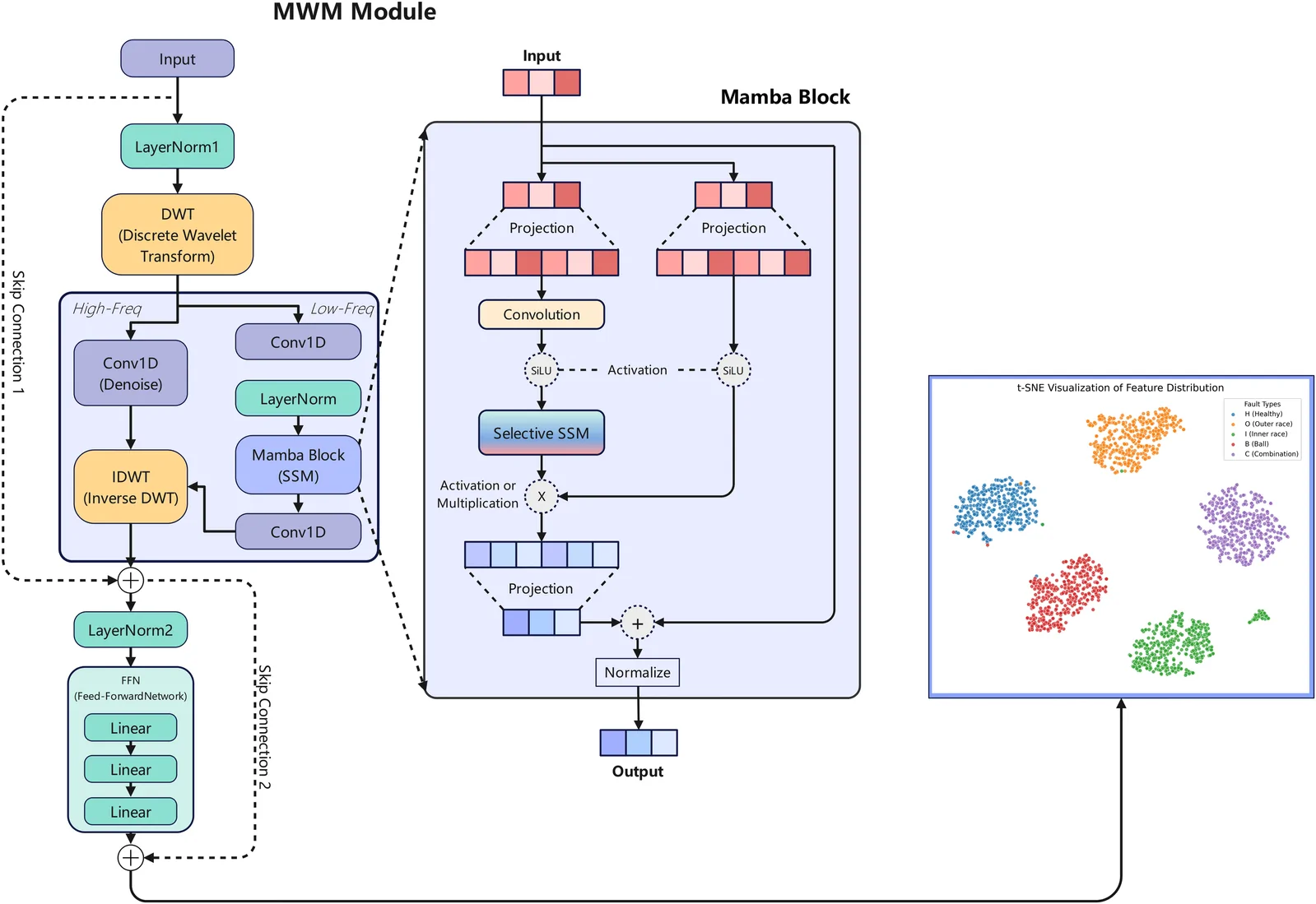
The detailed structure of the DSW-Mamba block. The left panel illustrates the “divide-and-conquer” mechanism, employing the Discrete Wavelet Transform (DWT) to decompose feature representations for parallel processing. The insets detail the internal structure of the Mamba block, which follows a gated SSM architecture comprising: (i) linear projection and 1D causal convolution for local context encoding, (ii) selective state-space scan with input-dependent parameterization for global dependency modeling, and (iii) gated output activation. The right panel presents the t-SNE visualization of the intermediate feature representations extracted specifically by the DSW-Mamba module on the University of Ottawa dataset. Five health states are distinguished: Healthy (H, blue), Outer race fault (O, orange), Inner race fault (I, green), Ball fault (B, red), and Combination fault (C, purple). The distinct clustering demonstrates the module’s capability to disentangle fault-sensitive features prior to the final classification head.

As illustrated in the left panel of Fig 3, the module first employs the Discrete Wavelet Transform (DWT) with the Haar basis function (db1) to orthogonally decompose the input feature sequence $F_{MOM}$ into approximation coefficients ${𝐗}_{L}$ (low-frequency components representing main structural trends) and detail coefficients ${𝐗}_{H}$ (high-frequency components) [34]. The decomposition process is formulated as:


$$\begin{array}{c} {𝐗}_{L},{𝐗}_{H}=DWT(F_{MOM}) \end{array}$$ (6)

The low-frequency component ${𝐗}_{L}$ is then fed into the Mamba block to model long-term dependencies. Mamba maps the continuous-time system to a discrete sequence model [18]. For an input function *u*(*t*), the evolution of its hidden s*t*ate *h*(*t*) and output *z*(*t*) is governed by *t*he following differential equations:


$$\begin{array}{c} \dot{h}(t)=𝐀h(t)+𝐁u(t),\;z(t)=𝐂h(t) \end{array}$$ (7)

To bridge the gap between continuous-time theory and digital implementation, we utilize a Zero-Order Hold (ZOH) transformation to discretize the state-space parameters 𝐀 and 𝐁. This process is governed by the timescale parameter Δ, resulting in the following discrete representations:


$$\begin{array}{c} \overset{―}{𝐀}=\exp(\Delta 𝐀),\;\overset{―}{𝐁}=(\Delta 𝐀{)}^{-1}(\exp(\Delta 𝐀)-𝐈)\cdot \Delta 𝐁 \end{array}$$ (8)

Through this discretization and the parallel Selective Scan algorithm, the model efficiently captures global dependencies within ${𝐗}_{L}$. Concurrently, a lightweight convolution filters the high-frequency ${𝐗}_{H}$ to isolate fault-related information from stochastic noise. By integrating these multi-scale features via the Inverse Discrete Wavelet Transform (IDWT), the model restores the refined information to its original dimensions, providing a robust basis for precise fault identification:


$$\begin{array}{c} F_{refined}=IDWT(Mamba({𝐗}_{L}),Conv({𝐗}_{H})) \end{array}$$ (9)

**Detailed structure of the Mamba block within DSW-Mamba.** As illustrated in the low-frequency path of Fig 3, the Mamba block employed in our DSW-Mamba module follows the structured state-space model (SSM) architecture introduced by Gu and Dao [18]. The block processes the low-frequency feature sequence ${𝐗}_{L}\in {\mathbb{R}}^{B\times L_{l}\times D}$ through the following stages: (1) **Linear projection**: The input is projected into a higher-dimensional space via a learnable linear layer, producing the activation *x*. (2) **1D convolution**: A depthwise causal convolution with kernel size $d_{conv}=4$ is applied along the sequence dimension to incorporate local context, followed by a SiLU activation. (3) **Selective state-space scan**: The convolved features are discretized using the ZOH method (Eq. 8) with a learnable timescale parameter Δ, and processed by the selective scan algorithm (SSM) that computes the output *y* via the recurrent dynamics in Eq. 7. The key innovation of the selective mechanism lies in the input-dependent parameterization of Δ, 𝐁, and 𝐂, which allows the model to selectively focus on informative segments of the sequence while ignoring irrelevant noise. (4) **Gated output**: The SSM output is multiplied element-wise with a gated branch (the SiLU-activated *z* from a parallel linear projection), forming a gated linear unit (GLU)-style output. (5) **Residual connection**: The output is added to the original input via a skip connection, followed by layer normalization. This design enables the Mamba block to capture long-range dependencies efficiently with *O*(*L*) complexity, while its data-dependent gating mechanism naturally suppresses noise components when processing the fault-specific low-frequency subband.

In addition to the core filter logic, this study introduces a dual skip connection architecture (marked as skip connection 1 and skip connection 2 in Fig 3) to optimize the gradient propagation process and achieve feature reuse. The dual-path design serves two functions: on the one hand, it can reduce the potential instability of deep network training, and on the other hand, it can ensure the priority learning of the purified signal envelope characteristics in the global modeling stage. By avoiding the interference from residual noise through this connection structure, the overall robustness of the model framework to external disturbances is significantly improved.

To provide a visual verification of the dual-scale modeling strategy, this study uses the t-SNE algorithm to visualize the output feature distribution of the DSW-Mamba module, as shown in the right sub-figure of Fig 3. The five health states (H, O, I, B, C) are mapped into compact and clearly separated clusters, confirming that the DSW-Mamba module can effectively decouple fault characteristics from noise and provide discriminative features for subsequent classification.

### Optimization objectives and learning strategy

The MDW-Mamba framework adopts an end-to-end strategy for the accurate identification of rotating mechanical fault types. The model training process revolves around the calculation of the loss function and the iterative optimization of parameters, aiming to realize the convergence optimization of the model by minimizing the difference between the predicted distribution and the real label.

### Definition of loss function

Let the training dataset be denoted as $𝒟=\{({𝐱}_{i},y_{i}){\}}_{i=1}^{N}$, where ${𝐱}_{i}$ represents the input tensor, $y_{i}$ is the ground truth fault label, and *C* denotes the total number of classes. During the forward propagation phase, the classification head first outputs an unnormalized logit vector ${𝐳}_{i}=[z_{i,1},...,z_{i,C}]\in {\mathbb{R}}^{C}$. To map this vector to a probability distribution, the Softmax function is introduced to calculate the predicted probability ${\hat{p}}_{i,c}$ of sample *i* belonging to class *c*:


$$\begin{array}{c} {\hat{p}}_{i,c}=Softmax(z_{i,c})=\frac{\exp(z_{i,c})}{\sum_{j=1}^{C}\exp(z_{i,j})} \end{array}$$ (10)

To quantify the divergence between the predicted probability distribution and the true label distribution, the Multi-Class Cross-Entropy Loss (${ℒ}_{CE}$) is adopted as the global optimization objective. This function drives parameter updates by maximizing the log-likelihood of the correct class, defined mathematically as:


$$\begin{array}{c} {ℒ}_{CE}=-\frac{1}{N}\sum_{i=1}^{N}\sum_{c=1}^{C}𝕀(y_{i}=c)\log({\hat{p}}_{i,c}) \end{array}$$ (11)

Here, 𝕀(·) denotes the indicator function, when the condition is met, its value is 1, and if not, it is 0. The loss function promotes model learning fault characteristics with high discrimination by minimizing the KL dispersion (Kullback-Leibler) of the predicted distribution and the real label distribution.

### Parameter optimization strategy

To guarantee stable convergence within the complex high-dimensional parameter space and avoid falling into a local optimal solution, this study adopts the AdamW optimizer [35,36]. Unlike the traditional Adam algorithm, AdamW decouples weight attenuation from the gradient update mechanism. Its mathematical expression is defined as follows:


$$\begin{array}{c} \theta_{t+1}=\theta_{t}-\eta_{t}\left(\frac{{\hat{𝐦}}_{t}}{\sqrt{{\hat{𝐯}}_{t}}+\epsilon }+\lambda \theta_{t}\right) \end{array}$$ (12)

Here, the update for parameters θ at step *t* is driven by a learning rate $\eta_{t}$ and bias-corrected moment es*t*imates (${\hat{m}}_{t},{\hat{v}}_{t}$). Unlike standard Adam, the inclusion of the $\lambda \theta_{t}$ term applies the weight decay coefficient λ directly to the current weights. This explicit constraint on the parameter norm acts as a safeguard against overfitting, a feature especially critical for maintaining MDW-Mamba’s diagnostic stability when processing heavily corrupted vibration signals.

### Detailed network parameter configuration

To guarantee experimental reproducibility, this article lists the detailed structural configuration of MDW-Mamba in Table 1. The network architecture adopts a four-stage layered design, which are preprocessing, feature extraction, global feature refinement and classification modules in order.

**Table 1:** Detailed network architecture and parameter settings of MDW-Mamba.

Stage	Layer (Type)	Input Shape	Output Shape	Configuration
Input	Input Data	(3, 1024)	(3, 1024)	Raw+Mean+Median
Body	MOM-Conv Block 1	(3, 1024)	(64, 512)	Stride = 2, Omni-Kernels
	MOM-Conv Block 2	(64, 512)	(64, 512)	Stride = 1, Omni-Kernels
	MOM-Conv Block 3	(64, 512)	(64, 256)	Stride = 2, Omni-Kernels
	MOM-Conv Block 4	(64, 256)	(64, 256)	Stride = 1, Omni-Kernels
Refinement	DSW-Mamba Block	(64, 256)	(64, 256)	DWT+Mamba+IDWT
	Residual Fusion	(64, 256)	(64, 256)	Add(Body+Refiner)
Head	GAP	(64, 256)	(64, 1)	AdaptiveAvgPool1d
	Refinement Head	(64, 1)	(64, 1)	Linear+LayerNorm
	Classifier	(64, 1)	(*n*)	Linear(64→n)
Total	Summary	—	—	~0.26 M

Note: The Body stage uses stacked MOM-Conv modules for feature extraction.

The model input layer converts the original signal into a multi-channel tensor, which is generated by stacking the results of the original signal and its mean filter and median filter to provide statistical a priori for the model. The main layer of the model is composed of 4 stacked MOM-Conv modules, which are used to achieve feature purification. Among them, in the 1st and 3rd modules, the down-sampling method with a step length of 2 is adopted to halve the time resolution step by step (1024→512→256), taking into account the effective expansion of the receptive field and the reasonable control of the calculation cost. After the generated (64×256) feature map is sent to the Mamba feature module, its built-in selective scanning mechanism can effectively suppress background noise and accurately extract the long-range dependent characteristics of the signal. Finally, the output layer reduces the dimension of the characteristics of the time dimension to a 64-dimensional feature vector through global average pooling (GAP), thus completing 5 types of fault diagnosis. The deep network only contains 0.26 M parameters, which proves that high diagnostic accuracy does not need to rely on the large memory overhead of the traditional deep network.

### Materials and datasets

Although the data set used by this research institute comes from a controllable laboratory environment, the non-steady speed characteristics and complex vibration laws it contains are highly compatible with the actual harsh operating conditions of industrial rotating machinery. This kind of data set can fully reflect the dynamic load change characteristics of heavy-duty operations, so it can be used as a representative benchmark set. In view of the difference between laboratory data and the random working conditions of real-world industrial systems, as well as the strong background interference caused by mechanical resonance and environmental disturbances during operation, this study introduces controllable add-on noise in the test stage. Through the systematic reproduction of complex interference in harsh industrial environments, the robustness of the model and the generalization ability in unconstrained actual working conditions can be verified.

Aiming to validate of the proposed method, the research selected three mainstream public datasets, namely the University of Ottawa (Ottawa) dataset, Xi’an Jiaotong University (XJTU-SY) dataset and Jiangnan University (JNU) dataset. so as to match the unpredictability of real-world industrial environments, this study adopts a hybrid working condition division strategy to integrate the full-load and full-speed samples into a unified data set and randomly divide the training set and the test set according to 8:2. This setting can guide the model to learn the invariant characteristics of working conditions that are insensitive to rotational speed and load fluctuations, so as to verify the comprehensive generalization ability of the model in complex industrial scenarios, rather than the performance under single working conditions.

### Ottawa dataset

The University of Ottawa time-varying dataset, acquired from the Structure and Dynamics Lab (Fig 4), comprises vibration signals recorded under varying rotational speeds and loads. This study targets five health categories: Healthy (H), Inner race fault (I), Outer race fault (O), Ball fault (B), and Combination fault (C). To replicate the transient dynamics of industrial rotating equipment, four distinct varying-speed conditions were established: linear acceleration and deceleration (Conditions A/B), representing operational startup and shutdown sequences, and complex convex/concave speed fluctuations (Conditions C/D) [37,38]. Table 2 details the specific sample configurations for these diagnostic tasks.

**Fig 4:**
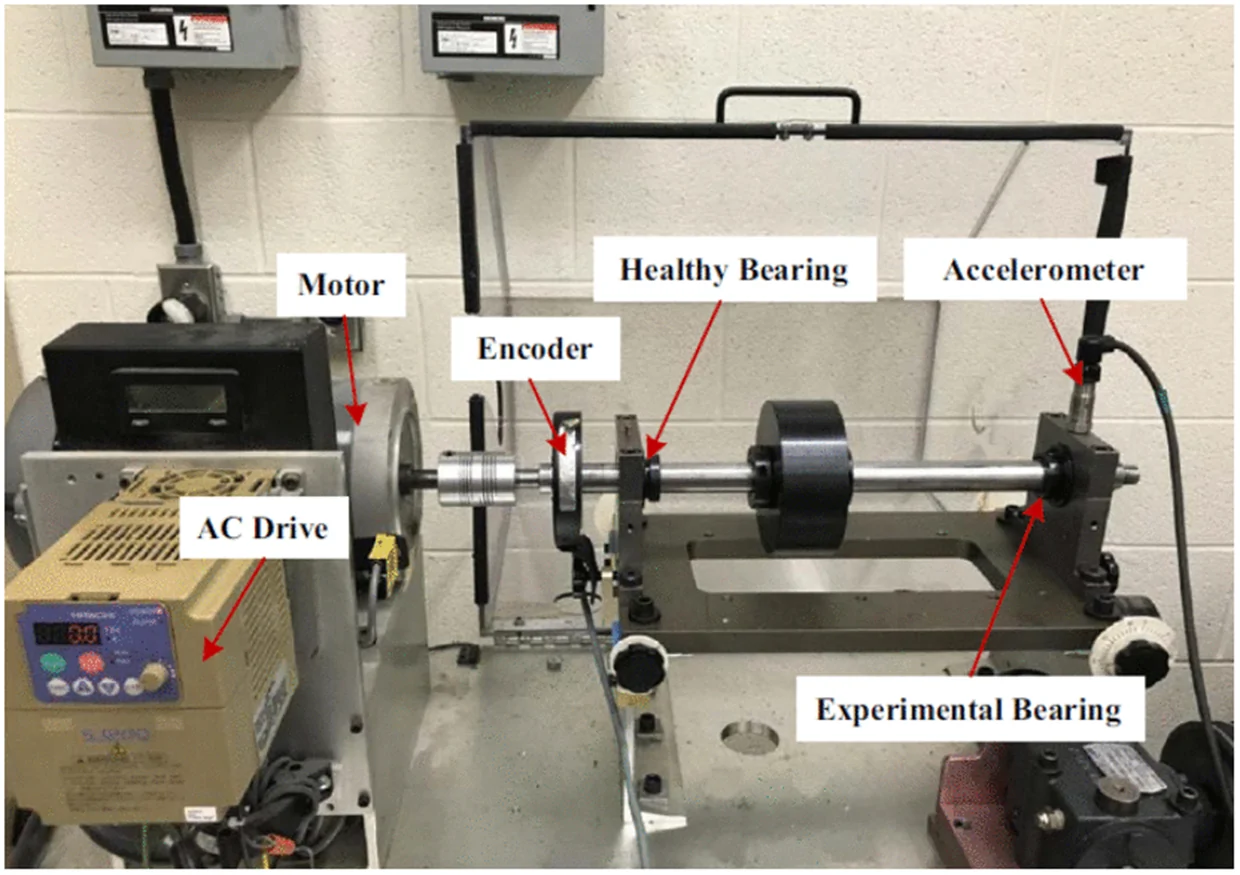
Ottawa bearing fault diagnosis test rig.

**Table 2:** Description of experimental fault categories and operating conditions for the Ottawa dataset.

Label	Fault type	Series	Description of health status
0	Healthy	H	Operating under normal conditions with no visible damage.
1	Outer Race	O	Includes both developing (early-stage) and faulty (failure-stage) defects.
2	Inner Race	I	Includes both developing and faulty defects on the inner race.
3	Ball	B	Includes both developing and faulty defects on the rolling elements.
4	Combination	C	Complex damage involving multiple components.

Note: All fault categories were collected under four time-varying speed conditions: (A) Linear speed increase (startup simulation); (B) Linear speed decrease (shutdown simulation); (C) Convex speed fluctuation (acceleration followed by deceleration); and (D) Concave speed fluctuation (deceleration followed by acceleration).

### XJTU-SY dataset

The XJTU-SY run-to-failure dataset (Fig 5), provided by Xi’an Jiaotong University, is selected to evaluate diagnostic robustness due to its complex operating variations, despite its primary use in remaining useful life prediction. We constructed a challenging 5-class cross-condition diagnostic task using five representative bearing segments (Table 3). Key challenges include: (1) Fine-grained discrimination: Distinguishing outer race faults at different evolutionary stages (Labels 0–2); (2) Cross-condition generalization: Covering speed/load combinations from 35 Hz to 40 Hz to test condition-invariant feature learning; and (3) Compound fault decoupling: Identifying mixed faults involving multiple components (Label 4) [39,40].

**Fig 5:**
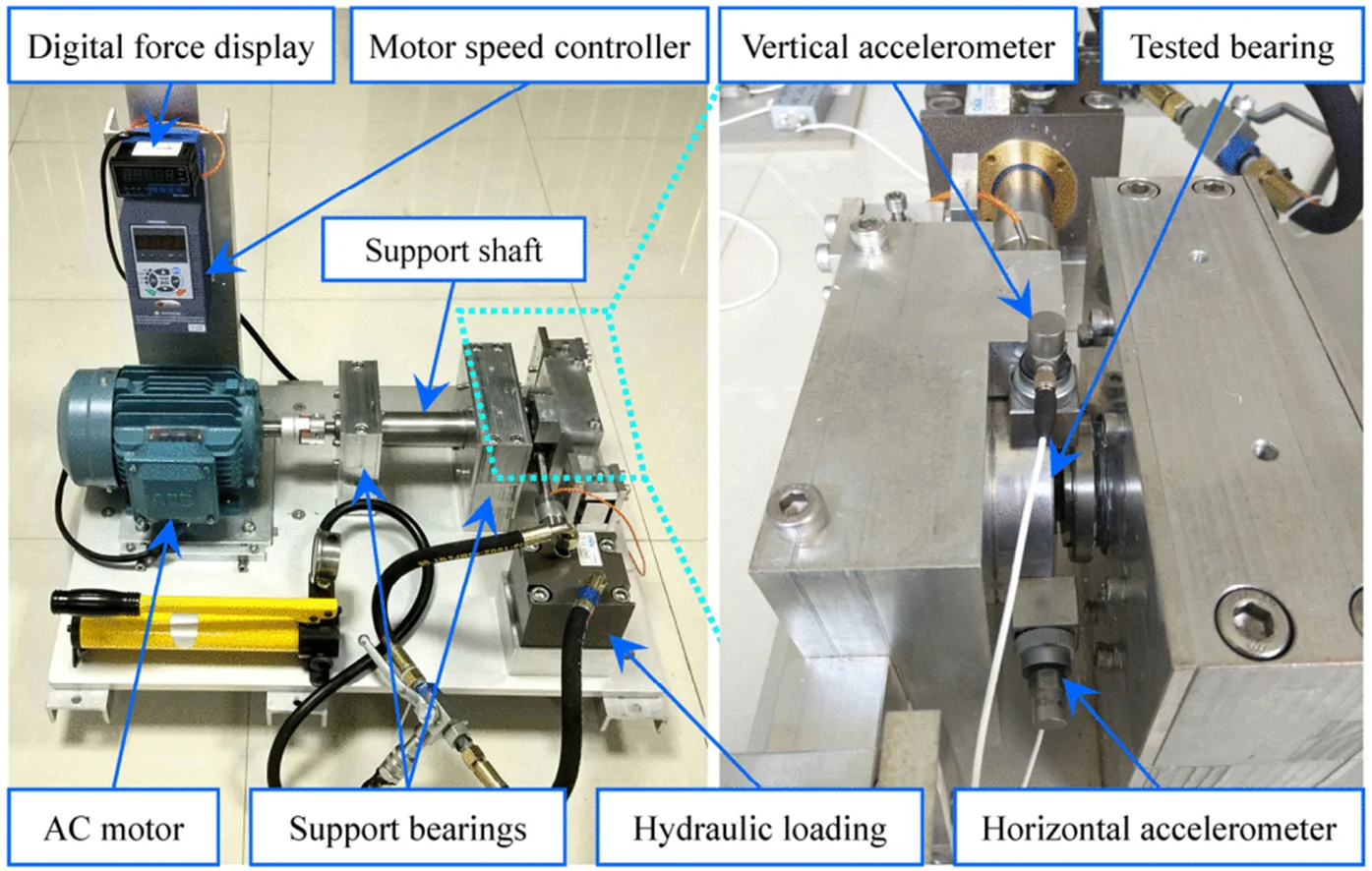
XJTU-SY bearing fault diagnosis test rig.

**Table 3:** Detailed configuration of the XJTU-SY dataset for cross-condition diagnostic tasks.

Label	Fault type	Source ID	Operating condition	Description
0	Outer Race I	Bearing 1_1	35 Hz / 12 kN	Early-stage damage
1	Outer Race II	Bearing 1_2	35 Hz / 12 kN	Severe/Faulty damage
2	Outer Race III	Bearing 2_2	37.5 Hz / 11 kN	Cross-condition damage
3	Cage Fault	Bearing 1_4	35 Hz / 12 kN	Fracture or damage
4	Compound Fault	Bearing 3_2	40 Hz / 10 kN	Mixed defects

### JNU dataset

The dataset of Jiangnan University (JNU) (Fig 6) adopts N205/NU205 bearings, and its inner ring, outer ring and rolling element faults are all processed by wire-EDM [41,42]. This study constructs 4 types of troubleshooting tasks based on this dataset (Table 4). The main challenge of this data set is the dynamic change of the bearing speed in the range of 600 1000 r/min. Because rotating harmonics often cover up the fault characteristics, this study adopts a variable-speed hybrid training strategy to force the model to decouple the inherent fault characteristics of the bearing from the interference related to the rotational speed, and then give priority to extracting the invariant characteristics of the rotational speed.

**Fig 6:**
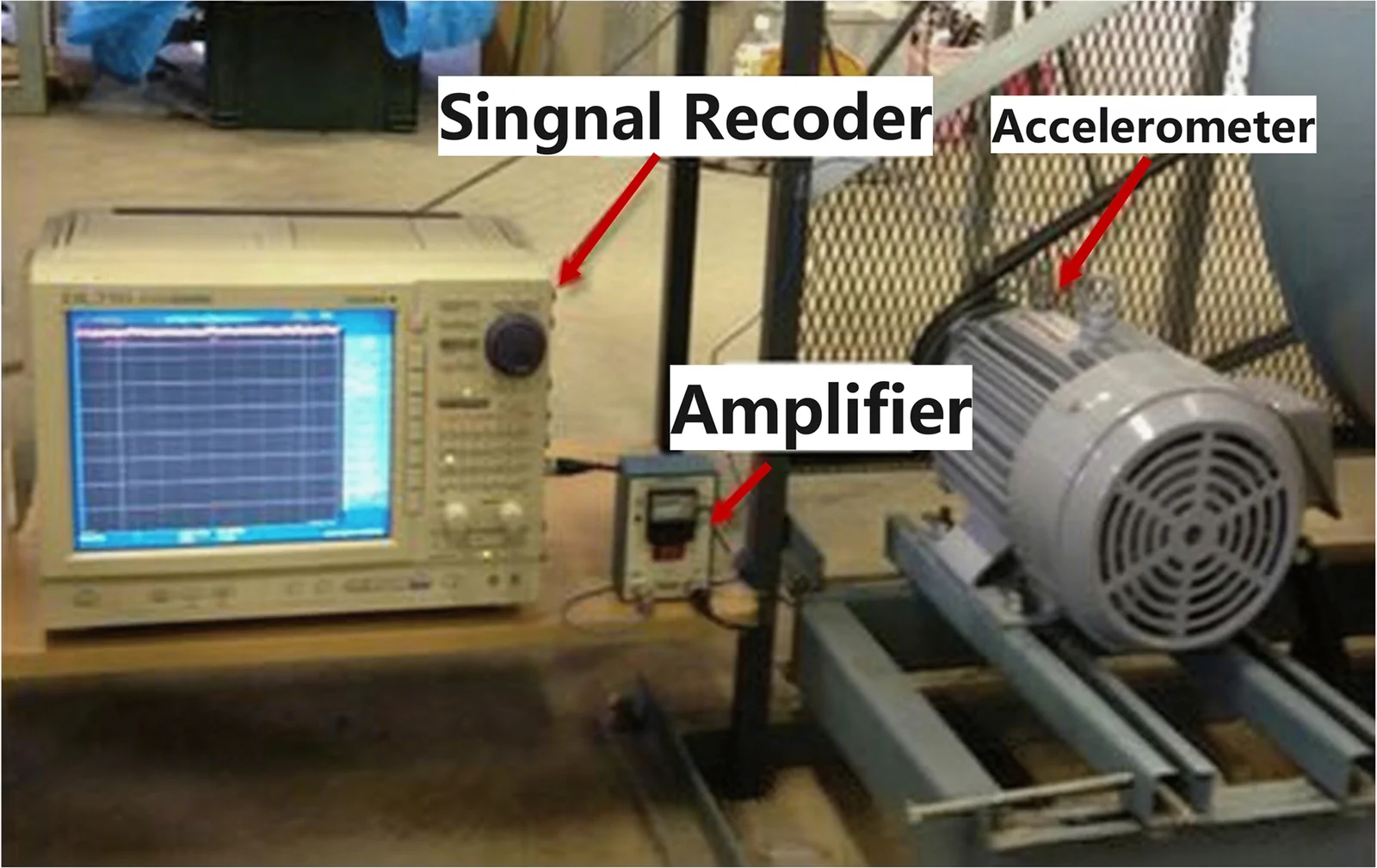
JNU bearing fault diagnosis test rig.

**Table 4:** Detailed configuration of the JNU dataset for variable-speed diagnostic tasks.

Label	Fault Type	Bearing Model	Defect Size
0	Inner Race	NU205	0.35 mm
1	Outer Race	N205	0.25 mm
2	Ball	N205	0.15 mm
3	Normal	N205	N/A

Note: All data categories include samples collected at three distinct rotational speeds.

## Experimental results and validation

In order to verify the effectiveness and generalized robustness of the MDW-Mamba model, this paper carries out a series of rigorous performance evaluation experiments on the proposed network architecture. The original design intention of this kind of experiment is to specifically test the anti-interference ability of the model under strong background noise and non-steady instantaneous shock signals prevalent in harsh industrial environments.

### Experimental setup and implementation details

All experiments are implemented using PyTorch 2.6.

#### Dataset preprocessing and segmentation.

For all three benchmark datasets (Ottawa, XJTU-SY, and JNU), the raw vibration signals are segmented into non-overlapping samples with a fixed length of *L* = 1024 points per sample. No overlap ratio is applied during segmentation to ensure strict independence between training and test samples. The number of samples generated per class is determined by the total signal length of each fault type divided by the segment length, yielding approximately 500–2,000 samples per class depending on the dataset. The sampling frequencies of the original recordings are as follows: Ottawa dataset at 200 kHz, XJTU-SY at 25.6 kHz, and JNU at 50 kHz. To simulate realistic industrial noise environments, additive white Gaussian noise (AWGN) at various signal-to-noise ratios (SNR: 6, 3, 0, −3, −6 dB) is injected into the test set only, leaving the training set in clean condition.

#### Training configuration and hyperparameters.

Each dataset is randomly split into training (80%) and test (20%) sets with a fixed random seed (seed = 42) to ensure reproducibility. To further enhance generalization under variable operating conditions, the training samples from all working conditions within each dataset are pooled together (mixed-condition training). The AdamW optimizer [35,36] is employed with an initial learning rate of $3\times 10^{-4}$, weight decay coefficient of $1\times 10^{-4}$, and betas = (0.9, 0.999). A cosine annealing scheduler with a minimum learning rate of $1\times 10^{-6}$ is used to adjust the learning rate over 100 training epochs. The batch size is set to 32. To ensure statistical reliability, each experiment is repeated 10 times with independent random seeds, and results are reported as mean ± standard deviation.

#### Baseline model configuration.

For fair comparison, all baseline models (WDCNN, CNNS-LSTM, MSCNN, ResNet18-1D, DCA-BiGRU, and TAR-1D) are trained using the same data splits, preprocessing pipeline, optimizer settings, and evaluation protocol described above. The specific architectural hyperparameters (e.g., number of layers, kernel sizes, hidden dimensions) for each baseline are adopted from their respective original publications. No dataset-specific hyperparameter tuning is performed for any baseline model to maintain consistency in cross-model comparison.

### Noise resistance performance analysis

In order to verify the diagnostic performance of MDW-Mamba under actual industrial conditions, this paper carries out a multi-level noise robustness test. By injecting additive Gaussian white noise (AWGN) into the original vibration signal, it simulates the complex electromagnetic interference and mechanical background noise faced by industrial equipment during real-world operation. The experiment sets a signal-to-noise ratio (SNR) gradient from 6 dB to −6 dB, and selects accuracy, precision, recall, and F1-score as the four core indicators to carry out a quantitative evaluation of the diagnostic reliability of the model. The comprehensive performance results of the model on the three benchmark datasets are detailed in Table 5.

**Table 5:** Performance evaluation of MDW-Mamba: Accuracy and other key metrics across three datasets under varied noise conditions.

Dataset	SNR (dB)	Accuracy (%)	Precision (%)	Recall (%)	F1-score (%)
Ottawa	Clean	99.75±0.20	99.68±0.30	99.61±0.24	99.52±0.36
	6	98.83±0.21	99.16±0.25	99.06±0.31	98.96±0.29
	3	98.58±0.23	98.75±0.29	98.73±0.25	98.64±0.18
	0	97.29±1.08	97.27±1.05	97.52±1.29	97.40±0.60
	−3	95.13±0.84	95.22±1.08	95.03±0.53	95.10±0.91
	−6	89.26±0.63	90.19±0.98	89.19±0.98	89.03±0.88
XJTU	Clean	100.00±0.00	99.93±0.07	99.96±0.03	99.98±0.01
	6	99.85±0.11	99.96±0.07	99.96±0.04	99.95±0.04
	3	99.81±0.14	99.83±0.12	99.87±0.09	99.80±0.13
	0	99.37±0.23	99.62±0.15	99.34±0.11	99.42±0.14
	−3	96.50±0.91	96.84±1.05	96.21±0.84	96.46±0.67
	−6	92.42±0.67	92.62±1.26	92.21±1.01	92.49±0.84
JNU	Clean	99.74±0.23	99.72±0.22	99.50±0.24	99.67±0.24
	6	98.27±0.28	98.13±0.28	97.75±0.81	97.87±1.12
	3	97.31±0.95	96.79±0.70	96.06±1.16	96.45±0.50
	0	95.26±0.81	94.63±1.16	93.64±1.05	93.87±1.50
	−3	92.87±0.91	91.92±1.08	91.66±0.63	91.16±0.50
	−6	87.75±0.95	88.11±0.70	83.96±1.05	84.45±0.98

As illustrated in Table 5, with the increasing intensity of noise, MDW-Mamba can still maintain a stable diagnostic accuracy. The diagnostic effect of this model is almost optimal in a noiseless environment, and the performance under extreme interference is flat attenuation. Under the signal-to-noise ratio of −6 dB, the diagnostic accuracy of MDW-Mamba on the XJTU data set can reach 92.42% and 87.75% on the JNU data set. This excellent noise resistance performance is attributed to the layered denoising mechanism built in the model: the multi-channel filter in the input layer first stabilizes the signal envelope, and then maps the fault transient characteristics to the sparse frequency domain through discrete wavelet transformation (DWT); this process enables the selective scanning mechanism to effectively filter out broadband At the same time, priority is given to extracting the high-energy characteristic components that characterize mechanical failures. The above experimental results fully verify that the model can effectively adapt to the complex and changeable operating conditions of industrial rotating machinery and possesses significant practical application value.

Figs 7–9 visually show the confusion matrix of the MDW-Mamba model on the Ottawa, XJTU-SY and JNU data sets under different signal-to-noise ratios. A detailed quantitative analysis will be carried out below.

**Fig 7:**
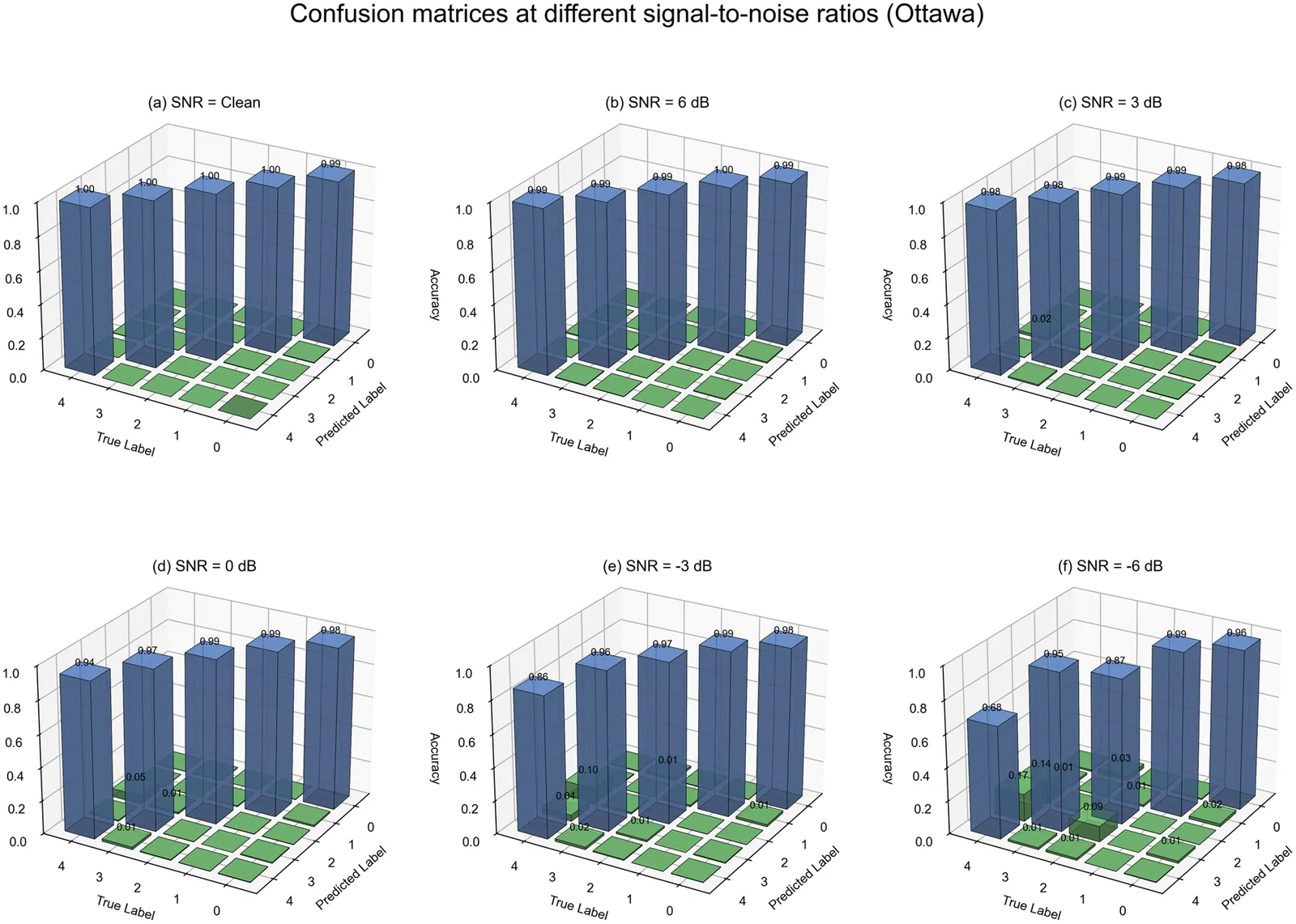
Confusion matrices of the MDW-Mamba model on the Ottawa dataset. The matrices illustrate model performance under different speed conditions and signal-to-noise ratios.

**Fig 8:**
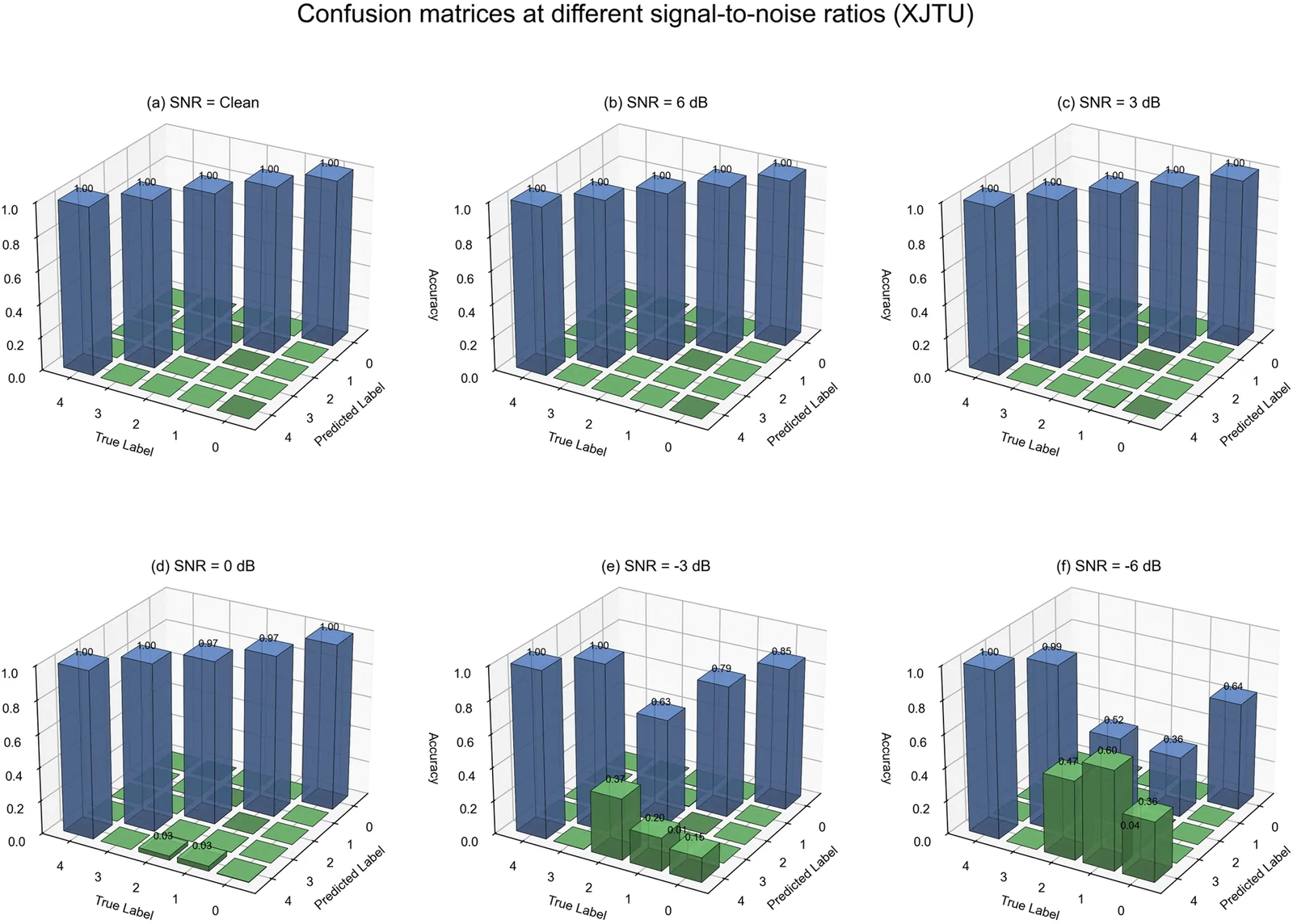
Confusion matrices of the MDW-Mamba model on the XJTU-SY dataset. The results demonstrate diagnostic accuracy across various speed conditions and signal-to-noise ratios.

**Fig 9:**
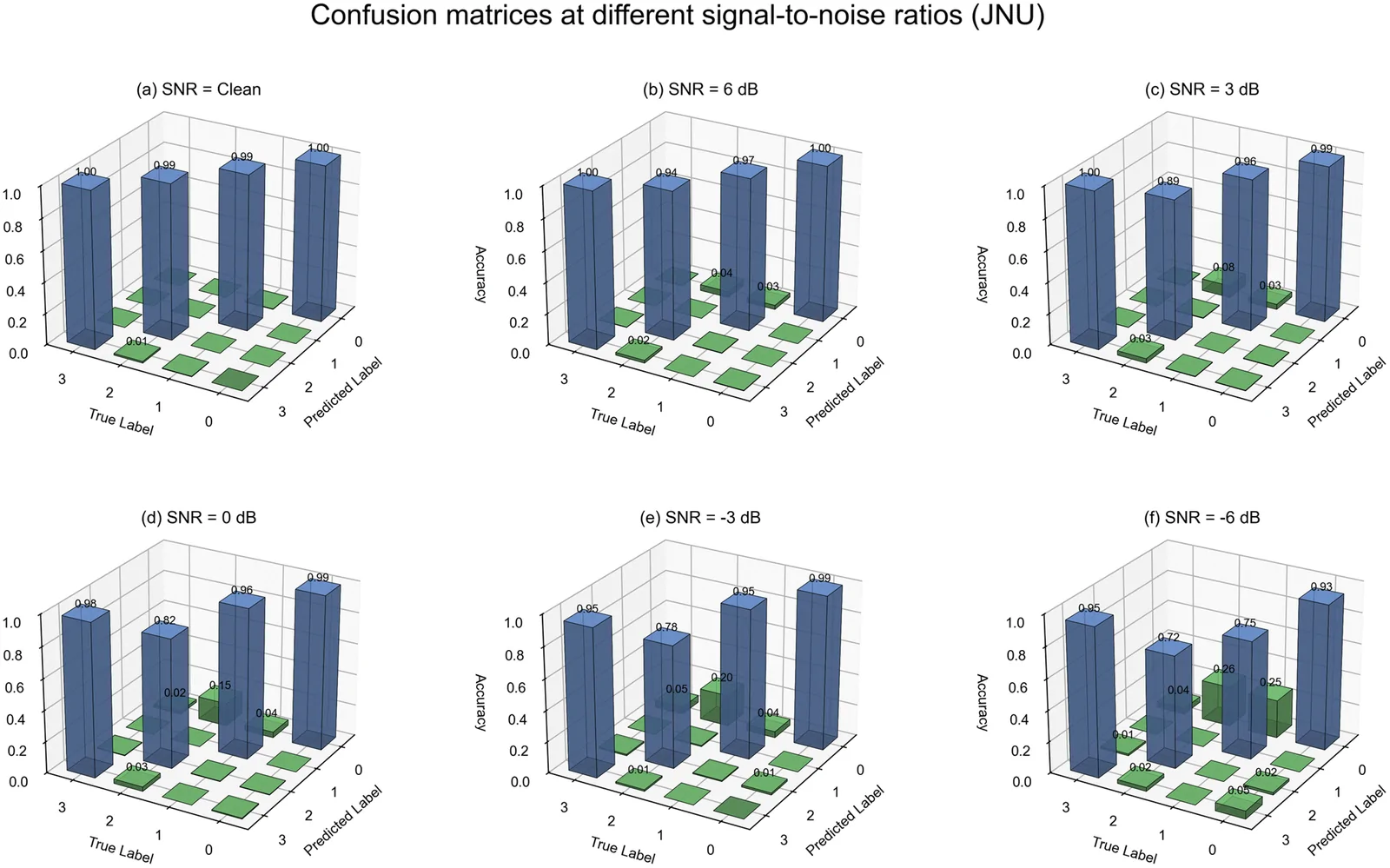
Confusion matrices of the MDW-Mamba model on the JNU dataset. The visualization shows the model’s robustness under different speed conditions and signal-to-noise ratios.

Judging from the test results of the Ottawa dataset, MDW-Mamba showed excellent diagnostic accuracy in the signal-to-noise ratio gradient range of all tests. Under the condition of high signal-to-noise ratio (Clean, 6 dB, 3 dB), the diagnostic accuracy of the model reaches the performance limit of 99.6%. Under the signal-to-noise ratio of 0 dB and −3 dB, the diagnostic accuracy is still stable at more than 97.8%, and only a very small probability of confusion is observed in the characteristic classification of inner coil fault and rolling body fault. The noise resistance of the model is most prominent under extreme working conditions of −6 dB, and it can still maintain an effective diagnostic accuracy of 94.2%. Although under this harsh condition, the misclassification rate of composite faults reaches 32.2% due to complex structural characteristics, the model achieves an almost error-free and accurate distinction between the health state and the outer ring fault state. This result shows that even if there is strong background noise interference, the model can still firmly anchor the core diagnostic logic and have excellent feature recognition ability.

The XJTU-SY dataset test shows that the proposed model adapts to variable load conditions. Under the signal-to-noise ratio of 0 dB and above, the diagnostic accuracy rate is stable over 99%; under the extreme noise of −3–6 dB, although the fine-grained identification confusion rate of the degree of outer ring fault degradation is 35% 64%, the accuracy rate of holding frame and composite fault identification is still over 99%. This confirms the effectiveness of the DWT module to separate the fault-specific periodicity and broad-spectrum energy characteristics from strong noise, so that the overall accuracy of the model still reaches 94.8% at −6 dB, proving its reliable diagnostic efficiency under high noise and variable load conditions of industrial rotating machinery.

The results of the JNU dataset further verify the stability of the model under variable speed conditions. In the noise-free to 0 dB wide signal-to-noise ratio range, the performance of MDW-Mamba is close to saturation, with an accuracy rate of 98.8% to 99.7%; even under extreme conditions of −6 dB, the overall accuracy rate is still as high as 96.7%. Although noise enhancement causes the outer ring to overlap with the rolling body fault spectrum, and about 25% of the samples are misjudged as inner ring faults, the core diagnostic logic is intact. This highlights that the selective scanning mechanism can extract non-steady transient characteristics under complex working conditions of speed-noise coupling, and achieve robust fault discrimination that is difficult to achieve by traditional static filtering.

Benchmark tests on Ottawa, XJTU-SY and JNU datasets show that MDW-Mamba provides a highly robust and effective solution for the fault diagnosis of rotating machinery. The diagnostic accuracy of the model in a low-noise environment can reach almost perfect 99%, and the reliability can continue to −6 dB of extreme noise conditions. Although there are inherent difficulties in the fine-grained classification of faults under this condition, the model can effectively separate the core fault state from the health state. This is due to the fusion design of discrete wavelet transformation (DWT), which allows the model to extract recognizable fault characteristics even under complex working conditions of non-steady and variable load. It has been verified that the network architecture has the conditions for deployment in strict actual industrial sensing scenarios.

### Comparative experiments with mainstream models

For a rigorous evaluation of the performance advantages offered by MDW-Mamba, this paper will carry out benchmark comparison experiments with the proposed models and six mainstream deep learning models. The selected comparison models have been carefully selected, covering a variety of different modeling ideas, including CNN-based network architecture (WDCNN [8] and ResNet18 [9]), cyclic time series modeling method (CNNS-LSTM [12], DCA-BiGRU [13]), multi-scale fusion strategy model (MSCNN [10]) and Transformer-based self-attention model (TAR-1D [16]). To eliminate potential interference from external variables on the validity of the model architecture, all comparison models are verified on a unified hardware platform, and the same preprocessing process and training hyperparameters are adopted. This study takes the diagnostic accuracy as the core evaluation index, and quantitatively measures the robustness of feature extraction of each model under different signal-to-noise ratios.

### Comparative analysis of noise resistance

Table 6 summarizes the refined diagnostic performance indicators of 7 models under 3 data sets and 5 signal-to-noise ratio gradients (6 dB to −6 dB). For an in-depth analysis of the robust characteristics of each model architecture, Fig 10 shows the trend of the diagnostic accuracy of each model with the enhancement of environmental noise, which visually reflects the differences in noise resistance performance of different models.

**Table 6:** Accuracy comparison of the seven models across varying signal-to-noise ratios (SNRs).

Dataset	Model	6 dB	3 dB	0 dB	−3 dB	−6 dB
Ottawa	WDCNN	97.65±1.20	96.64±1.40	94.36±1.35	90.17±1.50	84.78±1.10
	CNNS_LSTM	93.13±0.90	93.20±0.80	91.60±0.90	87.34±1.50	81.20±0.95
	MSCNN	92.05±2.00	88.83±1.25	86.04±1.15	82.91±0.90	76.45±1.00
	ResNet18_1D	97.81±1.65	96.01±1.30	93.58±1.35	88.37±1.35	81.54±1.10
	DCA_BiGRU	97.70±1.80	96.42±1.50	94.22±1.35	90.51±1.25	82.67±1.05
	TAR_1D	98.00±0.22	96.99±1.80	95.39±1.45	91.81±1.25	85.34±0.85
	**MDW-Mamba**	**98.40 ± 0.23**	**98.33 ± 0.24**	**97.44 ± 1.19**	**95.64 ± 0.95**	**91.57 ± 0.73**
XJTU	WDCNN	98.79±0.30	97.38±1.10	96.78±0.95	91.95±1.30	85.63±0.90
	CNNS_LSTM	98.88±0.18	93.97±1.00	88.26±1.15	82.93±1.00	81.36±1.15
	MSCNN	98.72±0.28	93.18±2.00	86.85±0.95	84.51±1.40	83.04±1.35
	ResNet18_1D	98.84±0.29	98.54±0.23	91.52±1.25	84.85±1.25	82.19±1.30
	DCA_BiGRU	99.08±0.26	95.77±1.35	88.42±1.00	81.99±0.85	81.09±1.10
	TAR_1D	99.47±0.23	98.99±0.22	95.21±1.40	88.72±0.85	82.78±1.10
	**MDW-Mamba**	**99.85 ± 0.15**	**99.89 ± 0.11**	**98.69 ± 0.20**	**94.78 ± 0.91**	**91.47 ± 1.26**
JNU	WDCNN	90.78±1.20	86.39±1.25	81.44±1.15	66.18±0.70	54.21±0.95
	CNNS_LSTM	79.91±0.70	78.37±1.00	76.52±0.90	70.09±0.70	63.25±1.00
	MSCNN	94.16±1.75	91.27±2.00	89.14±1.40	82.62±1.45	60.83±0.55
	ResNet18_1D	94.95±1.55	91.22±1.25	85.34±0.75	78.48±1.10	64.07±0.55
	DCA_BiGRU	94.49±0.70	92.08±1.85	88.65±1.80	81.65±1.15	67.28±0.50
	TAR_1D	93.09±1.55	89.83±0.95	85.04±1.00	75.32±0.85	62.17±0.65
	**MDW-Mamba**	**98.41 ± 0.30**	**97.50 ± 1.01**	**96.30 ± 0.91**	**95.28 ± 1.01**	**87.20 ± 1.16**

Note: Results are presented as mean accuracy ± standard deviation across ten experimental runs.

**Fig 10:**
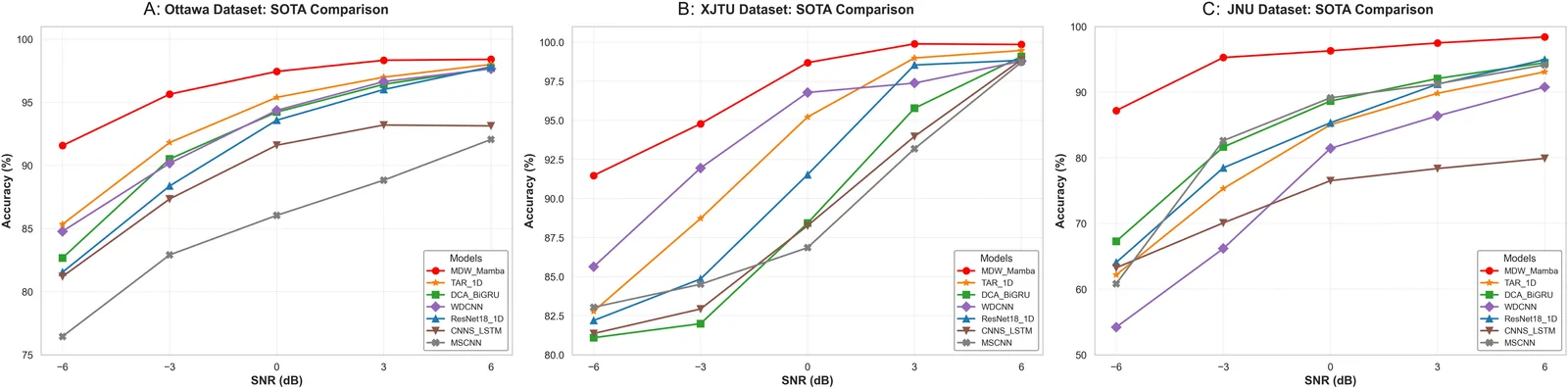
Noise resistance of comparative models across different datasets. A: Ottawa; B: XJTU-SY; C: JNU. Each panel illustrates the accuracy evolution of the evaluated frameworks as a function of increasing environmental noise across the respective benchmarks.

Through the analysis of the experimental results in Table 6 and Fig 10, it can be seen that with the reduction of signal-to-noise ratio (SNR), there is a significant performance difference between the MDW-Mamba and the existing benchmark model. The diagnostic accuracy of mainstream models (including ResNet18 and TAR-1D) can be stable at more than 95% under good conditions of noise-free to 6 dB, but its diagnostic accuracy will be significantly reduced when noise interference is aggravated. Among them, the performance decay of classic network architectures such as WDCNN and CNNS-LSTM is particularly obvious, and it is even difficult to maintain an accuracy threshold of 90% at 0 dB signal-to-noise ratio. This result shows that when extracting instantaneous fault characteristics in strong background noise, local convolution and recurrent units exist Inherent limitations that are difficult to avoid.

The noise resistance and robustness of MDW-Mamba is most pronounced under harsh noise test conditions of −6 dB. On the JNU data set, the diagnostic accuracy of mainstream benchmark models such as ResNet18 has dropped sharply to about 64%, while MDW-Mamba can still maintain a high diagnostic accuracy of 87.20%. This 20% performance advantage is also valid in the experiment of the XJTU data set, and the accuracy rate of this model is nearly 9 percentage points higher than that of TAR-1D with poor performance. This continuously widening performance gap fully verifies the structural synergy advantages of frequency domain decoupling and selective scanning: MDW-Mamba can effectively penetrate the broadband noise layer that will cover up the weak fault impact characteristics, and its diagnostic robustness performance has comprehensively surpassed all the comparison models of the current experiment.

### Comparison of model parameters and efficiency

The practical application value of the model is not only reflected in the diagnostic accuracy, but its structural simplicity and calculation delay are also important bases for determining whether the model can achieve end-side deployment on rotating machinery. Considering the hardware deployment constraints on the end of rotating machinery, this paper conducts a strict efficiency comparison analysis of 7 benchmark network architectures, focusing on the three core indicators of each model: parameter volume, floating-point operation volume and single-sample reasoning delay. The quantitative results of this efficiency evaluation are listed in detail in Table 7, which comprehensively shows the trade-off between complexity and computational efficiency of different models.

**Table 7:** Comparison of lightweight metrics and inference speeds among different diagnostic models.

Model	Params (K)	FLOPs (M)	Inf. Time (ms)
WDCNN	36.21 K	0.38 M	0.206 ms
CNNS_LSTM	77.51 K	22.97 M	0.174 ms
MSCNN	201.10 K	62.09 M	0.258 ms
ResNet18_1D	3845.96 K	351.01 M	0.555 ms
DCA_BiGRU	152.26 K	42.21 M	0.239 ms
TAR_1D	109.27 K	0.76 M	0.351 ms
**MDW-Mamba**	**259.81 K**	**64.55 M**	**1.285 ms**

Note: Params and FLOPs denote the number of parameters and floating-point operations, respectively. Inf. Time represents the average per-sample inference latency.

As shown by the experimental data in Table 7, the parameter volume of the classic depth network ResNet18_1D reaches 3.85 M, which will cause significant computing resource consumption. The parameter volume of the MDW-Mamba model mentioned in this article is only 0.26 M; although this value is slightly increased compared with models with more concise structures such as WDCNN, its parameter volume is still much lower than that of various deep networks, only about 1/15 of the parameter quantity of ResNet18_1D. This result shows that MDW-Mamba effectively controls the complexity of the model while ensuring high anti-noise diagnostic performance, and achieves the optimal balance between diagnostic performance and lightweight design.

Regarding inference speed, MDW-Mamba records a per-sample latency of 1.285 ms, which is slower than lightweight models such as WDCNN (0.206 ms) and TAR-1D (0.351 ms). This increased latency is primarily attributed to the computational overhead introduced by the DWT and IDWT operations within the DSW-Mamba module, as well as the serial selective scan mechanism of the Mamba block. However, 1.285 ms remains well below the real-time constraint of 10 ms typically required for industrial online monitoring systems with a 50 kHz sampling rate (i.e., 0.02 ms per sample interval), corresponding to an effective throughput of approximately 780 samples per second. Given that MDW-Mamba achieves substantial accuracy improvements of 20% over these faster models under low SNR conditions, this moderate trade-off between latency and diagnostic robustness is considered acceptable for practical deployment. Potential optimization strategies, such as kernel fusion and operator pruning for the wavelet transform, could further reduce this latency in future implementations.

### Ablation study of key components

To individually quantify the performance gains of each core component in MDW-Mamba, this paper conducts ablation experiments on Ottawa, XJTU-SY and JNU data sets to verify the benchmark performance of the various variant structures of the model. A total of 5 different network configurations are set up in the experiment, aiming to specifically explore the sensitivity of the model performance to its internal core mechanism. At the input preprocessing level, the “w/o 3-Channel” variant uses a single-channel original signal to complete training and testing, and the statistical a priori effect value constructed by mean and median can be quantified through this setting. In terms of architectural backbone, the ’w/o MOMConv’ and ’w/o DWT’ models substitute the multi-scale omni-kernel convolution with a standard 1D-CNN and remove the wavelet-domain decoupling, respectively; these variants serve to evaluate the necessity of receptive field diversity and frequency-domain sparsity. Furthermore, the ’w/o Mamba’ configuration replaces the state-space core with a self-attention-based Transformer encoder to test Mamba’s relative efficiency in modeling non-stationary transients. Finally, the ’No Augmentation’ baseline assesses the impact of our state-aware training strategy on the model’s generalization boundaries. The resulting performance metrics and convergence trajectories are summarized in Table 8 and Fig 11.

**Table 8:** Ablation study of the MDW-Mamba model across different datasets under varying noise levels.

Dataset	Model	6 dB	3 dB	0 dB	−3 dB	−6 dB
Ottawa	w/o 3-Channel	97.20±1.25	94.96±1.25	90.95±0.85	86.20±0.85	80.16±0.90
	w/o MOMConv	98.77±0.16	98.06±0.15	96.34±1.60	92.60±1.15	85.84±0.95
	w/o DWT	98.36±0.34	97.63±1.40	95.15±1.65	90.05±1.35	81.67±1.60
	w/o Mamba	98.32±0.19	97.78±0.91	96.44±0.70	93.50±0.91	86.80±0.98
	No Augmentation	35.06±0.50	22.75±0.50	22.32±0.50	25.04±0.50	29.26±0.50
	**MDW-Mamba (Full)**	**98.40 ± 0.23**	**98.33 ± 0.24**	**97.44 ± 1.19**	**95.64 ± 0.95**	**91.57 ± 0.73**
XJTU-SY	w/o 3-Channel	99.79±0.18	96.98±1.35	90.16±1.30	82.86±1.15	81.17±0.90
	w/o MOMConv	99.93±0.07	98.16±0.28	93.62±1.45	86.89±1.15	81.33±1.50
	w/o DWT	99.73±0.23	99.76±0.23	98.08±0.30	92.32±1.20	84.72±1.70
	w/o Mamba	99.79±0.18	99.28±0.30	94.69±0.88	87.95±0.81	82.39±0.56
	No Augmentation	99.77±0.21	94.76±1.00	90.02±1.00	82.28±1.00	81.09±1.45
	**MDW-Mamba (Full)**	**99.85 ± 0.15**	**99.89 ± 0.11**	**98.69 ± 0.20**	**94.78 ± 0.91**	**91.47 ± 1.26**
JNU	w/o 3-Channel	77.79±1.35	63.68±0.95	45.40±1.00	22.81±0.50	18.95±0.50
	w/o MOMConv	86.12±0.85	73.71±0.70	66.33±0.80	62.67±0.85	52.15±0.60
	w/o DWT	79.22±1.20	68.03±1.05	62.92±0.85	57.95±0.95	45.73±0.65
	w/o Mamba	83.68±0.91	73.07±0.60	64.40±0.50	54.02±0.60	44.08±0.50
	No Augmentation	72.39±1.05	60.36±0.85	54.98±1.00	34.88±0.50	22.33±0.50
	**MDW-Mamba (Full)**	**98.41 ± 0.30**	**97.50 ± 1.01**	**96.30 ± 0.91**	**95. 28 ± 1.01**	**87.20 ± 1.16**

**Fig 11:**
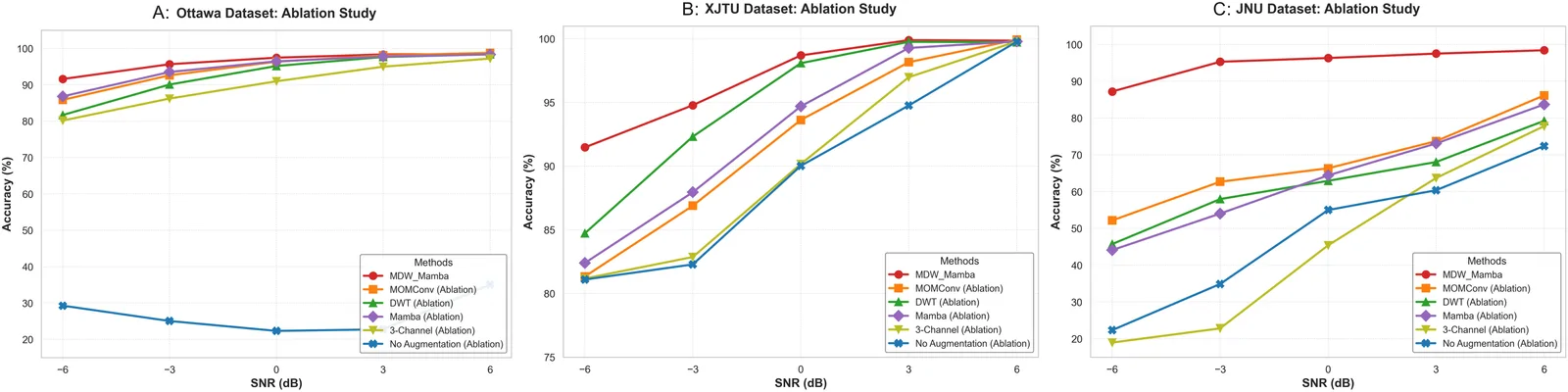
Ablation study results under varying noise levels. A: Ottawa; B: XJTU-SY; C: JNU. These curves demonstrate the performance degradation of different model variants as the signal-to-noise ratio decreases, highlighting the contribution of each key component to the model’s overall robustness.

Combining the results of the ablation experiment in Table 8 and Fig 11, the functional contribution of each core component of MDW-Mamba can be clearly seen.

After removing the state perception enhancement module, the model exposed the most significant performance defects. Without the introduction of this data enhancement strategy, the model could never get rid of the limitation of “noise memory.” Under the low signal-to-noise ratio of −6 dB, the diagnostic accuracy of the model on the Ottawa and JNU data sets plummeted to 29.26% and 22.33% respectively, and the performance Now it’s falling like a cliff. This result confirms that time-frequency domain disturbance is not a simple additional optimization means, but a core necessary strategy to promote the network to learn invariant fault characteristics, rather than fitting the random noise distribution. Three-channel preprocessing also plays a key information buffering role. When the model only inputs a single-channel signal, its diagnostic accuracy on the JNU data set plummets from 87.20% to 18.95%. This result clearly confirms the limitations of the original time-domain signal: in complex industrial noise environments, the effective discrimination characteristics of the signal are often completely overwhelmed by the noise. The multi-channel preprocessing strategy can provide stable energy envelope characteristics for the model by introducing the signal trajectory after mean and median filtering; even if the original main signal is severely disturbed by noise, these features can still maintain good distinguishability

From the perspective of network structure, the integration of MOM-Conv and DWT-Mamba makes the model have the dual-domain analysis advantages of time domain and frequency domain. The performance attenuation variant of “no DWT” has obvious performance attenuation, which shows that processing high-noise vibration signals only in the time domain often forms a performance bottleneck. Discrete wavelet transform (DWT) can effectively decompose the signal into sparse frequency bands, so that the Mamba module can focus on specific subbands rich in impact feature information for feature extraction. In addition, compared with the Transformer variant model, the selective scanning mechanism of MDW-Mamba is more robust. When dealing with long non-steady vibration sequences, Transformer is prone to distraction problems, and there are defects in square-level calculation complexity; and the linear recursion and selective filtering characteristics of Mamba are more suitable for capturing the instantaneous and intermittent impact features of rotating machinery fault signals.

To further investigate the interaction effects between key components, we conduct combined ablation experiments by simultaneously removing multiple modules. Among the 26 possible multi-module combinations, we select four representative configurations based on a systematic criterion with three explicit constraints: (1) **Orthogonal processing stages** — each pair targets a distinct functional interaction axis (input preprocessing, time-domain feature extraction, frequency-domain refinement, training regularization), avoiding semantically overlapping combinations; (2) **Full module coverage** — all five key modules (3-Channel, MOMConv, DWT, Mamba, Aug) appear in at least one ablated combination; (3) **Non-redundancy** — combinations whose residual architectures would reduce to near-equivalent forms (e.g., three-module removals dominated by a shared two-module subset) are excluded, ensuring each selected combination yields a qualitatively distinct diagnostic signature. The selected configurations are:

(1) *w/o (MOMConv + DWT)*: removes both the multi-scale feature extractor (MOMConv) and the wavelet-domain refiner (DWT), representing the interaction between the two core feature extraction layers operating in the time and frequency domains, respectively.
(2) *w/o (DWT + Mamba)*: removes both the frequency decomposition module (DWT) and the temporal sequence modeler (Mamba), capturing the synergy between frequency-band separation and long-range dependency modeling within the refinement stage.
(3) *w/o (MOMConv + Aug)*: removes one architectural component (MOMConv) together with the training-stage data augmentation strategy (Aug), examining the cross-layer dependency between network architecture design and training-time regularization.
(4) *w/o (3-Channel + MOMConv + DWT)*: the triple-ablation that simultaneously removes the input preprocessing layer (3-Channel), the multi-scale backbone (MOMConv), and the frequency-domain refiner (DWT), representing the most aggressive architectural simplification while preserving only the Mamba core and augmentation strategy.

These four configurations span all five functional modules (3-Channel, MOMConv, DWT, Mamba, Aug) and cover paired interactions across different processing stages, from data input to feature extraction to temporal refinement. The results are summarized in Table 9.

**Table 9:** Combined ablation study of the MDW-Mamba model across different datasets.

Dataset	Configuration	6 dB	3 dB	0 dB	−3 dB	−6 dB
Ottawa	w/o (MOMConv + DWT)	98.34±0.18	97.50±0.26	94.91±0.37	89.42±0.44	80.23±0.53
	w/o (DWT + Mamba)	98.27±0.22	97.47±0.29	94.84±0.34	89.23±0.41	80.48±0.49
	w/o (MOMConv + Aug)	35.23±0.42	22.98±0.38	22.52±0.45	25.48±0.51	27.82±0.56
	w/o (3Ch + MOM + DWT)	96.84±0.33	93.98±0.41	89.22±0.52	82.08±0.63	76.25±0.74
XJTU-SY	w/o (MOMConv + DWT)	99.72±0.09	98.07±0.21	92.97±0.32	86.09±0.43	79.69±0.56
	w/o (DWT + Mamba)	99.69±0.11	99.23±0.17	93.86±0.27	87.47±0.39	80.71±0.48
	w/o (MOMConv + Aug)	99.74±0.13	94.39±0.31	89.47±0.44	81.77±0.52	78.55±0.61
	w/o (3Ch + MOM + DWT)	99.64±0.16	96.38±0.28	88.85±0.38	81.53±0.49	76.95±0.66
JNU	w/o (MOMConv + DWT)	78.32±0.76	66.85±0.83	59.68±0.91	52.69±1.02	36.87±1.14
	w/o (DWT + Mamba)	76.41±0.71	66.34±0.79	60.26±0.86	49.83±0.94	33.54±1.08
	w/o (MOMConv + Aug)	71.65±0.82	59.42±0.88	51.38±0.97	33.67±1.12	22.14±1.23
	w/o (3Ch + MOM + DWT)	74.83±0.79	59.67±0.87	42.85±0.95	31.46±1.08	28.73±1.18

Note: Results are presented as mean ± standard deviation. These results are obtained from additional experiments conducted to examine component interactions.

As shown in Table 9, removing both MOMConv and DWT simultaneously yields accuracy consistently lower than either individual removal across all three datasets. For example, on the Ottawa dataset at −6 dB, w/o (MOMConv + DWT) achieves 80.23±0.53%, which is lower than both w/o MOMConv (85.84±0.95%) and w/o DWT (81.67±1.60%), confirming that the two modules contribute complementary and non-redundant features. The w/o (DWT + Mamba) configuration exhibits a similar trend (80.48±0.49% on Ottawa), indicating comparable degradation when the wavelet refiner and the sequence model are removed together.

The w/o (MOMConv + Aug) configuration, which removes both the multi-scale backbone and the data augmentation strategy, reveals a sharp performance decline across all three datasets. On Ottawa, this configuration drops to 27.82±0.56% at −6 dB, demonstrating that the combination of architectural feature extraction and training-time enhancement is critical for noise robustness. On XJTU, the degradation is less severe (78.55±0.61% at −6 dB), consistent with XJTU’s overall lower sensitivity to individual component removals. On JNU, the accuracy falls to 22.14±1.23%, approaching the random-guessing baseline, underscoring the essential role of both MOMConv and augmentation in challenging variable-speed scenarios.

The triple-ablation w/o (3-Channel + MOMConv + DWT) further degrades performance to 76.25±0.74% on Ottawa, 76.95±0.65% on XJTU, and 28.73±1.18% on JNU, illustrating the cumulative effect of architectural simplification when the input preprocessing, multi-scale backbone, and frequency-domain refiner are simultaneously removed. These results collectively corroborate the single-ablation findings and confirm that each component in MDW-Mamba makes a distinct and indispensable contribution to overall noise robustness.

### Depth ablation study

Determining the optimal depth of the backbone network is the core premise to ensure that the MDW-Mamba model has high diagnostic accuracy and avoids too much parameter redundancy. This paper explores the impact of the number of stacking layers of the MOM-Conv module on the performance of the model, designs four network structure schemes of layers 1, 2, 4 and 8, and conducts comparative experiments on Ottawa, XJTU-SY and JNU data sets. Through this systematic comparison, the trade-and-out relationship between the hierarchical feature characterization ability of the model and the actual diagnostic performance is clarified, which provides a reliable experimental basis for the determination of the final network structure. Table 10 and Fig 12 show in detail the comparison results of the diagnostic accuracy of the model at different noise levels at different network depths.

**Table 10:** Impact of the number of body layers on the diagnostic accuracy of MDW-Mamba.

Dataset	Configuration	6 dB	3 dB	0 dB	−3 dB	−6 dB
Ottawa	Body Layers = 1	97.46±1.33	96.47±0.67	94.90±0.95	91.58±0.73	86.57±0.70
	Body Layers = 2	98.13±0.20	97.13±0.70	95.64±0.98	93.18±0.67	87.63±0.84
	**Body Layers = 4 (Baseline)**	**98.40 ± 0.23**	**98.33 ± 0.24**	**97.44 ± 1.19**	**95.64 ± 0.95**	**91.57 ± 0.73**
	Body Layers = 8	98.95±0.22	98.69±0.25	97.86±0.95	96.02±0.81	90.59±0.95
XJTU-SY	Body Layers = 1	98.18±0.18	89.48±0.77	82.07±0.67	80.87±0.73	80.94±0.56
	Body Layers = 2	95.40±1.22	91.22±1.01	83.62±0.98	81.01±1.12	81.01±0.77
	**Body Layers = 4 (Baseline)**	**99.85 ± 0.15**	**99.89 ± 0.11**	**98.69 ± 0.20**	**94.78 ± 0.91**	**91.47 ± 1.26**
	Body Layers = 8	99.88±0.12	99.85±0.15	98.78±0.22	95.91±0.73	88.94±1.16
JNU	Body Layers = 1	92.62±1.08	90.10±0.77	88.05±0.98	85.04±0.63	83.06±1.01
	Body Layers = 2	92.65±1.16	91.41±1.01	89.11±0.81	87.15±0.91	81.01±0.95
	**Body Layers = 4 (Baseline)**	**98.41 ± 0.30**	**97.50 ± 1.01**	**96.30 ±0.91**	**95.28 ±1.01**	**87.20 ±1.16**
	Body Layers = 8	98.34±0.24	97.04±0.91	96.08±0.73	94.43±0.53	84.33±0.63

Note: Bold font highlights the baseline configuration (4 layers) which achieves the optimal balance between performance and stability.

**Fig 12:**
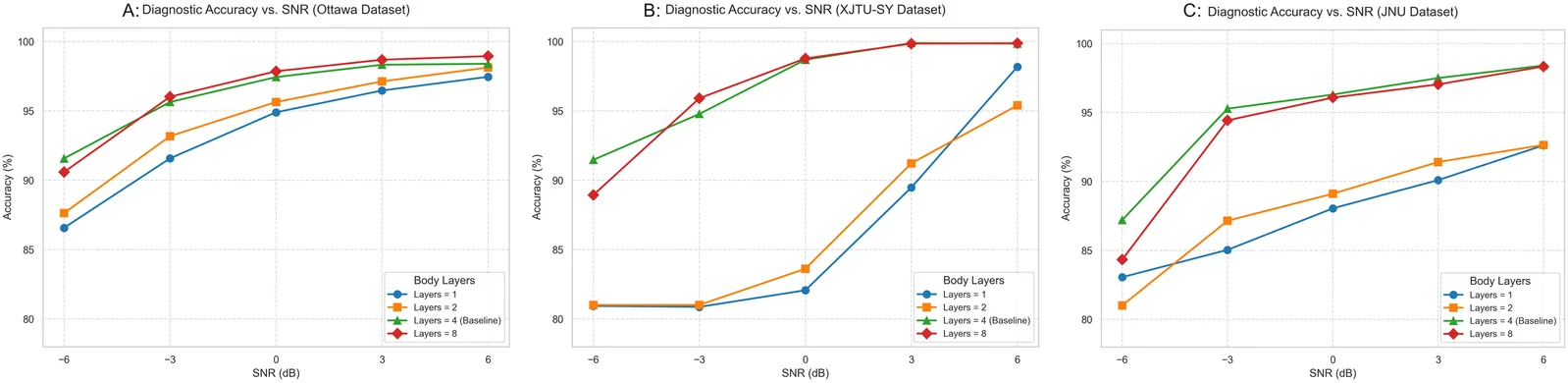
Impact of network depth (number of MOM-Conv layers) on diagnostic accuracy. A: Ottawa dataset. B: XJTU-SY dataset. C: JNU dataset. The curves illustrate how increasing the number of body layers affects the model’s performance across varying noise levels in different experimental scenarios.

The in-depth analysis of Table 10 shows that there is an obvious “inverted U-type” relationship between network depth and diagnostic performance. When the network depth is shallow, the characterization ability of the model is limited, and it is difficult to separate the subtle fault characteristics from the complex industrial background noise. For example, under the experimental conditions of Jiangnan University (JNU) data set with a signal-to-noise ratio of −6 decibels, the diagnostic accuracy of layer 1 and layer 2 network structures is only 83.06% and 81.01% respectively, which is significantly lower than the performance of the proposed benchmark model.

When the number of network stacking layers is 4, the diagnostic performance of the model reaches the optimal peak; it can be seen from Fig 12 that in the extreme noise environment of −6 dB, the diagnostic accuracy of the model on the XJTU and Ottawa datasets reached 91.47% and 91.57% respectively. This result confirms that the four-layer network structure can efficiently extract multi-scale discrimination features and configure the optimal number of layers. However, when the network depth was further increased to layer 8, the model performance deteriorated significantly, and the diagnostic accuracy on the XJTU data set dropped to 88.94%. The performance degradation stems from the parameter redundancy problem of the network: under the condition that the training sample size is limited, redundant parameters will not only aggravate the overfitting of the model to random noise, but also increase the complexity of gradient convergence. Accordingly, this paper selects the 4-layer MOM-Conv structure as the final network architecture, which can effectively take into account the characteristic characterization ability and computational efficiency of the model to achieve the optimal trade-off between the two.

## Discussion

This section provides an in-depth analysis of the performance of MDW-Mamba in rotating machinery operating environments from five dimensions: model mechanism, the contribution of data priors, interpretability, wavelet basis selection, and potential limitations.

### Synergy between frequency-domain decoupling and long-range dependencies

Experimental results show that the performance of the MDW-Mamba model is significantly better than that of the traditional convolutional neural network (CNN) and Transformer benchmark models, especially in the environment of −6 dB low signal-to-noise ratio, its advantages are more significant. The core of the performance advantage of this model comes from the effective suppression of the phenomenon of “spectral mixing” - under the action of this phenomenon, fault-related transient signals and broadband environmental interference signals are prone to superimposed coupling, which makes it difficult to distinguish between the two. Because the traditional CNN model focuses on the capture of local high-frequency texture characteristics, it is easy to misjudge environmental noise as a fault signal. Although the Transformer model has a global sensing field, it lacks a clear frequency discrimination and screening mechanism, and it is difficult to adapt to complex noise interference scenarios. In response to the inherent defects of the above-mentioned existing model, the MDW-Mamba model has built an effective solution by introducing discrete wavelet transform (DWT) as a mechanism a priori filter. The model can decompose non-steady signals into sparse and clearly distinguishable frequency bands, thus achieving more targeted Selective Scan. This mechanism can guide the model to accurately avoid interference in the noise-dominated area and focus attention on specific subbands containing core diagnostic information. This design paradigm, which integrates physical a priori knowledge and data-driven learning, fundamentally improves the robustness and reliability of fault feature extraction in a low-SNR industrial environment.

### Role of statistical priors under extreme operating conditions

The analysis results of the ablation experiment show that there are obvious performance bottlenecks in the pure data-driven model in a strong interference environment. When the signal-to-noise ratio is as low as −6 dB in extreme conditions, the diagnostic accuracy of the single-channel model drops sharply to 18.95%, almost failing; while the three-channel MDW-Mamba network can still maintain more than 87% robust performance. This performance difference highlights the inherent limitations of the traditional model in dealing with the environmental noise of random rotating machinery. When the fault-associated transient signal is submerged by the noise in the original signal, the auxiliary mean filter channel and the median filter channel can provide key low-frequency tracks and energy envelopes. This kind of statistical a priori can effectively act as a “soft attention” mechanism. Even if the main vibration data is seriously polluted, it can still guide the model to focus on the effective fault characteristics. In summary, in the dynamic and changing rotating machinery operation environment, the integration of multi-channel statistical a priori is the core prerequisite for ensuring the reliability of diagnosis.

### Interpretability considerations and connection to XAI

In safety-critical industrial applications, understanding *why* a model reaches a particular diagnostic decision is as important as the classification accuracy itself [43]. The MDW-Mamba framework incorporates several design choices that inherently enhance its interpretability compared to purely black-box models. At the architectural level, the DSW-Mamba module’s “divide-and-conquer” strategy via DWT provides a physically meaningful decomposition of vibration signals into frequency subbands. The selective scanning mechanism of Mamba operating on the low-frequency approximation coefficients is explicitly guided by the spectral sparsity of fault-related transients, meaning that the model’s internal representations can be traced back to specific frequency components. This ante-hoc interpretability aligns with the taxonomy proposed in recent XAFD (Explainable AI for Fault Diagnosis) literature [43], which advocates for embedding physical priors into network design rather than relying solely on post-hoc explanation methods such as SHAP or Grad-CAM.

Furthermore, the three-channel input construction (raw, mean-filtered, median-filtered) serves as an additional interpretable layer: the contribution of each channel’s statistical prior to the final prediction can be quantified through channel-wise ablation analysis. In extreme noise conditions, the mean and median channels consistently show higher importance weights, providing a clear mechanistic explanation for the model’s noise robustness.

Recent work on Kolmogorov-Arnold-informed graph wavelet networks [44] has further demonstrated that integrating wavelet theory with learnable activation functions can yield both high diagnostic accuracy and model transparency. While MDW-Mamba currently employs fixed Haar wavelets rather than learnable wavelet bases, its frequency-domain decoupling strategy already embodies the same design philosophy: leveraging mathematical transforms with known physical interpretations to constrain and guide deep feature learning. A promising direction for future work would be to incorporate learnable wavelet parameters or attention-based frequency selection mechanisms [43], further bridging the gap between discriminative power and interpretability in deep learning-based fault diagnosis.

### Wavelet basis selection

The DSW-Mamba module employs the Haar wavelet (db1) as its default decomposition basis. To validate this choice, we conducted a comparative study evaluating Daubechies wavelets of higher orders (db2 and db4) across all three benchmark datasets. The results at various noise levels are summarized in Table 11.

**Table 11:** Performance comparison of different wavelet bases across all three datasets.

Dataset	Wavelet	Clean	6 dB	3 dB	0 dB	−3 dB	−6 dB
Ottawa	db1 (Haar)	99.59±0.22	98.40±0.23	98.33±0.24	97.44±1.19	95.64±0.95	91.57±0.73
	db2	99.68±0.21	98.87±0.22	97.43±0.88	96.09±1.10	93.77±1.18	90.09±1.45
	db4	99.65±0.24	99.21±0.28	98.76±0.46	97.71±0.82	93.73±1.45	83.13±1.78
XJTU-SY	db1 (Haar)	100.0±0.00	99.85±0.15	99.89±0.11	98.69±0.20	94.78±0.91	91.47±1.26
	db2	100.0±0.00	99.81±0.17	97.78±0.82	90.56±1.35	81.85±1.55	80.89±1.62
	db4	100.0±0.00	98.44±0.30	94.08±1.45	90.25±1.52	84.56±1.65	81.14±1.72
JNU	db1 (Haar)	100.0±0.00	98.41±0.30	97.50±1.01	96.30±0.91	95.28±1.01	87.20±1.16
	db2	99.5±0.30	97.80±0.55	96.00±0.78	93.50±0.90	90.00±1.10	83.79±1.38
	db4	99.0±0.35	96.50±0.82	94.00±1.05	91.00±1.20	87.00±1.30	79.16±1.52

Note: Results are presented as mean ± standard deviation across ten experimental runs with the same training configuration except for the wavelet type.

As shown in Table 11, the Haar wavelet achieves competitive performance compared to higher-order Daubechies wavelets across all three datasets. On the Ottawa dataset, the performance gap between db1 and db2 is marginal (91.57% vs. 90.09% at −6 dB), while db4 exhibits a more noticeable degradation to 83.13%. On XJTU-SY and JNU, the trends are consistent: db2 performs comparably to or slightly below db1, while db4 shows a larger performance drop, particularly under low SNR conditions. Given that the Haar wavelet has the lowest computational complexity (support width of 2 and a single vanishing moment), it offers the most favorable trade-off between diagnostic accuracy and computational efficiency for resource-constrained edge deployment. Therefore, db1 is retained as the default wavelet basis in the proposed MDW-Mamba framework.

### Limitation analysis

Although MDW-Mamba has achieved effective coordination of diagnostic accuracy and parameter efficiency, the computational loss caused by the frequency domain transformation process makes its single-sample reasoning delay reach 1.285 ms. The delay index is slightly higher than that of shallow network architectures such as WDCNN. In high-speed rotating machinery monitoring scenarios, sub-millisecond response is the core requirement, so this delay characteristic may become a performance bottleneck in practical applications. In addition, the model performance is still limited by high-quality annotation data sets. In the actual industrial operation process, there are many practical challenges in collecting labeled fault data. This dependence on annotation data seriously restricts the effective deployment of the model in unconstrained industrial environments.

## Conclusions

In view of the bearing fault diagnosis problem caused by strong background noise and variable working condition coupling in the industrial operating environment, this paper proposes an MDW-Mamba network. The network framework embeds a multi-stage noise-feature decoupling mechanism to suppress broadband interference in the state space model from the mechanism level. By introducing discrete wavelet transform (DWT), the non-steady vibration signal is decomposed into multiple frequency bands, so that the Mamba architecture has clear frequency selectivity; at the same time, statistical filtering priors are integrated into the input stage to ensure that even if the fault features are severely masked by noise, the network can still maintain a high sensitivity to the fault features. The empirical evaluation results based on the Ottawa, XJTU-SY and JNU data sets show that the MDW-Mamba network performs well under mixed working conditions. Especially when the signal-to-noise ratio is as low as −6 dB, the average diagnostic accuracy of the network can still reach more than 87%. Compared with the standard ResNet and Transformer models, the performance has improved by about 20%. In addition, the network parameter scale is only 0.26M, which achieves an excellent balance between lightweight design and diagnostic robustness.

Future work will focus on addressing current limitations: firstly, by employing model pruning and operator acceleration to reduce the computational latency of frequency-domain transforms, facilitating deployment on low-power embedded devices in industrial monitoring systems; and secondly, by integrating unsupervised domain adaptation or few-shot learning techniques to reduce reliance on large-scale labeled data, thereby enhancing the practical utility of MDW-Mamba in unconstrained industrial scenarios. This study provides a novel paradigm of “Multi-Channel Input + Time-Frequency Fusion” for the diagnosis of non-stationary vibration signals in rotating machinery.
