# Supplementary material for: Robust bearing fault diagnosis for rotating machinery under noisy and variable operating conditions using a Wavelet-Mamba Network
Source: PLoS One. 2026 Sep 28;21(9):e0358759. doi: 10.1371/journal.pone.0358759 (PMC13618967; doi:10.1371/journal.pone.0358759)
Supplement: Supplementary file 1 [file pone.0358759.s001.pdf]

Response to Reviewers

Manuscript ID: PONE-D-26-00901

Title: Robust bearing fault diagnosis for rotating machinery under noisy and variable operating conditions using a wavelet-Mamba network

Journal: PLOS ONE

Dear Editor and Reviewers,

We sincerely thank the Academic Editor, Dr. Zhibin Zhao, and both reviewers for the thorough and constructive evaluation. We have carefully addressed every point raised. Below is our point-by-point response. All changes in the revised manuscript are marked with track changes.

Part I: Responses to Journal Requirements

J1. PLOS ONE Style Requirements

Comment: Please ensure that your manuscript meets PLOS ONE's style requirements, including those for file naming.

Response: Thank you for this reminder. The revised manuscript has been reformatted according to the PLOS ONE style templates. File naming follows the journal's conventions.

J2. Code Sharing

Comment: We expect all author-generated code to be made available without restrictions upon publication.

Response: Thank you for this important requirement. The complete source code for the proposed MDW-Mamba framework, including model architecture, training pipeline, evaluation scripts, and data preprocessing utilities for all three benchmark datasets (Ottawa, XJTU-SY, JNU), has been made publicly available on GitHub: `https://github.com/Newdawn01/mdw_mamba`. The repository includes a detailed README with setup instructions, usage examples, and hyperparameter configurations.

J3. Role of Funder Statement

Comment: Please state what role the funders took in the study.

Response: Thank you for this clarification request. The funders had no role in study design, data collection and analysis, decision to publish, or preparation of the manuscript. This amended statement is included in the cover letter.

J4. Data Availability Statement

Comment: The Data Availability Statement is currently missing the name of the third party contact or institution / contact details.

Response: Thank you for pointing out this omission. The Data Availability Statement has been updated with full contact information. All three datasets are publicly available: the Ottawa

dataset from the University of Ottawa (DOI: 10.1016/j.dib.2018.11.019); the XJTU-SY dataset from Xi'an Jiaotong University (DOI: 10.3901/JME.2019.16.001); and the JNU dataset from Jiangnan University (DOI: 10.1016/j.isatra.2020.08.010).

J5. Figures 8 and 9 References

Comment: Please ensure that you refer to Figures 8 and 9 in your text.

Response: Thank you for this technical reminder. Figures 8 and 9 are properly referenced in the text (Section 4.2.1). All figure cross-references have been verified.

J6. Supplementary Figure File Type

Comment: Please amend the file type of supplementary figures to 'Supporting Information'.

Response: Thank you for this reminder. We have carefully re-examined our submission. The 12 uploaded image files correspond to Figures 1–12 of the main manuscript, all cited and discussed in the manuscript body. No supplementary figures are included in this submission. All figures are therefore correctly categorized as 'Figure' type, consistent with PLOS ONE guidelines.

J7. Citation Recommendations

Comment: If the reviewer comments include a recommendation to cite specific previously published works, please review and evaluate these publications.

Response: Thank you for this guidance. We have carefully reviewed the recommended publications and found them directly relevant. The following works are now cited in the revised manuscript:

- Shi et al. (2024), IEEE TII, DOI: 10.1109/TII.2023.3345449
- Shi et al. (2023), Knowledge-Based Systems, DOI: 10.1016/j.knosys.2022.110172

Both references have been verified against their official metadata.

Part II: Response to Academic Editor

Editor Comment: The reviewer gave some useful comments, the author should revise the paper comprehensively before acceptance.

Response: We thank the Editor for this guidance. We have comprehensively revised the manuscript, addressing every comment from both reviewers. Key additions include: (1) a theoretical justification for Mamba's low-frequency modeling capability (Section 2.4); (2) detailed implementation specifications (Section 4.1); (3) combined ablation experiments (Section 4.3, new Table 9); (4) an interpretability discussion connecting to XAI literature (Section 5.3); and (5) a wavelet basis selection study (Section 5.4, new Table 10).

Part III: Response to Reviewer #1

We sincerely thank Reviewer #1 for the rigorous and expert evaluation. We have carefully addressed each comment below.

R1-1. Theoretical Basis for Mamba's Low-Frequency Adaptability

Comment: The author mentioned in Section 2.4 that although the computational complexity of the Mamba architecture has been reduced to a linear level, when directly applied to noisy high-frequency vibration signals, it is prone to overfitting. However, no theoretical basis has been provided for its adaptability to low-frequency signals. It is suggested to provide supplementary explanations.

Response: Thank you for identifying this theoretical gap. We have added a detailed explanation in Section 2.4. The justification draws from the state-space model (SSM) formulation: the continuous-time dynamics matrix $\mathbf{A}$ governs how past information propagates or attenuates, functioning as an implicit low-pass filter. The Zero-Order Hold discretization with timescale parameter Δ introduces a Nyquist-type constraint — frequencies exceeding $1/(2\Delta)$ are inherently aliased. When raw high-frequency signals with broadband noise are directly fed into Mamba, stochastic noise fluctuations force the state transition matrices to fit spurious high-frequency patterns, causing overfitting. Conversely, the low-frequency approximation coefficients $\mathbf{X}_L$ produced by DWT possess a compact spectral distribution matching the SSM's implicit bandwidth constraints, enabling stable long-range dependency modeling.

R1-2. Summary of Five Health States in Figure 3 t-SNE Visualization

Comment: It is suggested that a summary and explanation of the changes in the five different states be included in the visualization graph of the DSW-Mamba Block output results shown in Figure 3.

Response: Thank you for this practical suggestion. Figure 3 has been updated with explicit health state labels (H, O, I, B, C) and consistent color coding (blue, orange, green, red, purple) in the t-SNE panel. The figure caption identifies all five states and notes that the distinct clustering demonstrates the module's capability to disentangle fault-sensitive features. The accompanying text in Section 2.4 confirms that the five health states are mapped into compact and clearly separated clusters, indicating effective noise-feature decoupling.

R1-3. Mamba Block Principle Diagram and Explanation

Comment: The Mamba module is one of the important components in this article. However, the author does not provide a detailed explanation of the applicability and design purpose of the Mamba module in the text. It is suggested that the author add the principle diagram and related explanations of the Mamba Block within the DSW-Mamba module.

Response: Thank you for this recommendation. Figure 3 has been enhanced with an inset diagram detailing the internal structure of the Mamba block. We have added a comprehensive description in Section 2.4 elaborating the five-stage architecture: (1) Linear projection for feature expansion; (2) 1D causal convolution for local context encoding; (3) Selective state-space scan with input-dependent parameterization; (4) Gated output via element-wise multiplication with a SiLU-activated branch; and (5) Residual connection with layer normalization. The design purpose — capturing long-range dependencies with $O(L)$ complexity while selectively suppressing noise in the fault-specific low-frequency subband — is explicitly discussed.

R1-4. Inference Speed Explanation (Table 7)

Comment: In Table 7, the inference speed of this model is at the slowest level, but there is no explanation for this in the text.

Response: Thank you for this pertinent observation. We have added a quantitative analysis in Section 4.2.2. The per-sample latency of 1.285 ms is attributed to the DWT/IDWT operations and the serial selective scan mechanism. This latency remains well below the real-time constraint of 10 ms for industrial monitoring, corresponding to approximately 780 samples per second throughput. Given the approximately 20% accuracy improvement over faster models under low SNR, this moderate trade-off is acceptable. We also discuss potential optimization strategies including kernel fusion and operator pruning.

R1-5. Combined Module Ablation Study (Table 8)

Comment: Table 8 merely shows the impact of different individual modules on the overall performance of the model, without reflecting the performance of different combined modules on the overall model performance. This lacks sufficient persuasiveness.

Response: Thank you for this critical suggestion. We have conducted combined ablation experiments, presented in a new Table 9 (Section 4.3). Among the 26 possible multi-module combinations, four representative configurations were selected based on three explicit constraints: (1) Orthogonal processing stages — each pair targets a distinct functional interaction axis (input preprocessing, time-domain feature extraction, frequency-domain refinement, training regularization); (2) Full module coverage — all five key modules (3-Channel, MOMConv, DWT, Mamba, Aug) appear in at least one ablated combination; (3) Non-redundancy — configurations that would reduce to near-equivalent residual architectures are excluded. Results across all three datasets confirm that the five modules contribute complementary and non-redundant features, with simultaneous removal causing greater degradation than any single-module removal alone.

R1-6. Interpretability and Connection to XAI

Comment: The manuscript could benefit from a deeper discussion on the interpretability of the learned features. The authors are encouraged to discuss how their method aligns with recent developments in Explainable Artificial Intelligence (XAI) for fault diagnosis.

Response: Thank you for this forward-looking suggestion. We have added a new subsection "Interpretability Considerations and Connection to XAI" (Section 5.3). We discuss how MDW-Mamba embodies ante-hoc interpretability: the DSW-Mamba module's DWT-based decomposition provides physically meaningful frequency subband separation; the three-channel input construction enables channel-wise ablation to quantify each statistical prior's contribution; and the frequency-domain decoupling strategy aligns with the XAFD taxonomy proposed by Li et al. (Reliability Engineering & System Safety, DOI: 10.1016/j.ress.2025.111935). We also connect our work to the Kolmogorov-Arnold-informed graph wavelet networks recommended by the reviewer (Li et al., IEEE TSMC, DOI: 10.1109/TSMC.2025.3647102) and discuss learnable wavelet parameters as a future direction.

Part IV: Response to Reviewer #2

We sincerely thank Reviewer #2 for the meticulous and methodologically rigorous evaluation. We have carefully addressed each comment below.

R2-1. Missing Implementation Details

Comment: Important implementation details are missing or vague, such as sampling frequency, segment length generation, overlap ratio, number of samples per class, train/validation/test split, batch size, learning rate, weight decay value, number of runs, random seeds.

Response: Thank you for this thorough checklist. We have added a comprehensive "Experimental Setup and Implementation Details" subsection (Section 4.1) specifying every item raised:

- **Software framework**: PyTorch 2.6
- **Sampling frequencies**: Ottawa 200 kHz, XJTU-SY 25.6 kHz, JNU 50 kHz
- **Segment length**: $L = 1024$, no overlap (stride $= L$)
- **Samples per class**: approximately 500–2,000 depending on dataset and condition
- **Data split**: 80% training / 20% test, stratified random split, seed = 42
- **Noise injection**: AWGN applied to test set only; training on clean data
- **Training**: Mixed-condition training across all operating conditions
- **Optimizer**: AdamW, lr $= 3 \times 10^{-4}$, weight decay $= 1 \times 10^{-4}$, $\beta = (0.9, 0.999)$
- **Learning rate schedule**: Cosine annealing, minimum lr $= 1 \times 10^{-6}$
- **Epochs**: 100, batch size = 32
- **Number of runs**: 10 independent repetitions with different random seeds
- **Reported metrics**: Mean accuracy ± standard deviation across 10 runs

R2-2. Distinction from Prior Wavelet-Mamba Methods

Comment: The manuscript claims novelty through wavelet-guided Mamba and noise-feature decoupling, but the distinction from prior wavelet-Mamba or Mamba-based fault diagnosis methods needs to be sharpened.

Response: Thank you for this incisive comment. We have added an explicit comparative discussion in the Introduction, identifying two fundamental differences:

1. **Noise decoupling vs. feature enrichment**: Prior works (e.g., Yang et al., 2025; Lai et al., 2025) employ wavelets primarily for multi-resolution feature enrichment or multi-channel fusion, without addressing the physical separation of fault features from broadband noise. Our DSW-Mamba module implements a frequency-domain "divide-and-conquer" strategy where DWT orthogonally decomposes the signal, and Mamba selectively models only the low-frequency, fault-dominant components.
2. **Frequency-selective scanning**: Unlike existing Mamba-based methods that process full-band signals, MDW-Mamba's selective state-space scan operates exclusively on compact, DWT-decomposed low-frequency coefficients. This fundamentally prevents overfitting to high-frequency noise patterns. At SNR $= -6$ dB, MDW-Mamba achieves approximately 87% accuracy, surpassing competing models by approximately 20 percentage points.

This distinction is reinforced in the updated Contribution (2) statement.

R2-3. Low-Resolution or Cropped Figures

Comment: Several figures appear low-resolution or cropped, especially the architecture and comparison plots.

Response: Thank you for bringing this to our attention. All figures have been re-processed through the PLOS ONE NAAS (New Automated Article Submission) tool, which automatically standardizes image dimensions to journal specifications and verifies resolution compliance. All 12 figures now pass the NAAS technical compliance check with "Image complies with specification" status. The architecture diagram (Figure 1), comparison plots (Figure 10), and all other figures have been verified at 300 DPI or higher.

R2-4. Baseline Model Hyperparameter Configuration

Comment: The authors should clarify whether the same hyperparameters were used for all baseline models or tuned separately for each model.

Response: Thank you for this important question. We have added an explicit statement in Section 4.1: "All baseline models use the same data splits, preprocessing pipeline, optimizer settings, and evaluation protocol as MDW-Mamba. Architectural hyperparameters for each baseline model follow the specifications reported in their original publications. No dataset-specific or model-specific hyperparameter tuning was performed beyond the published configurations, ensuring a fair and reproducible comparison."

R2-5. Wavelet Basis Selection

Comment: The manuscript should explain why Haar wavelet/db1 was selected and whether other wavelet bases were tested.

Response: Thank you for this methodological suggestion. We have added a new subsection "Wavelet Basis Selection" (Section 5.4) with a comparative study across three Daubechies wavelet bases (db1/Haar, db2, db4). The results (Table 11) demonstrate that the Haar wavelet achieves competitive or superior performance to higher-order wavelets across all three datasets and SNR levels, while offering the lowest computational complexity (support width = 2, single vanishing moment). This makes db1 the optimal choice for resource-constrained edge deployment. All results are presented as mean ± standard deviation across ten experimental runs.

R2-6. References Updated with Recent Studies

Comment: References should be standardized and updated with more recent 2023–2026 studies, such as 10.1109/TII.2023.3345449, 10.1016/j.knosys.2022.110172.

Response: Thank you for these literature recommendations. We have updated the reference list with the recommended works and additional recent studies (2023–2026):

- Shi et al. (2024), IEEE TII, DOI: 10.1109/TII.2023.3345449
- Shi et al. (2023), Knowledge-Based Systems, DOI: 10.1016/j.knosys.2022.110172
- Li et al. (2026), Reliability Engineering & System Safety, DOI: 10.1016/j.ress.2025.111935
- Li et al. (2026), IEEE TSMC, DOI: 10.1109/TSMC.2025.3647102

All references have been verified against their official metadata and formatted according to Vancouver style.

We hope the revised manuscript now satisfactorily addresses all concerns. We remain at your disposal for any further questions.

Sincerely,

Dayou Cui, Zhaoyan Xie, Zhixue Wang, Xiaowei Li, Xiangxuan Meng
