# Supplementary material for: Robust bearing fault diagnosis for rotating machinery under noisy and variable operating conditions using a Wavelet-Mamba Network
Source: PLoS One. 2026 Sep 28;21(9):e0358759. doi: 10.1371/journal.pone.0358759 (PMC13618967; doi:10.1371/journal.pone.0358759)
Supplement: Peer review history

# Peer review: Decision Letter 0

**DOI:** 10.1371/journal.pone.0358759.r001

**Authors:** Zhibin Zhao

17 May 2026

Dear Dr. Xie,

Thank you for submitting your manuscript to PLOS ONE. After careful consideration, we feel that it has merit but does not fully meet PLOS ONE’s publication criteria as it currently stands. Therefore, we invite you to submit a revised version of the manuscript that addresses the points raised during the review process.

Please submit your revised manuscript by Jul 01 2026 11:59PM. If you will need more time than this to complete your revisions, please reply to this message or contact the journal office at plosone@plos.org. When you're ready to submit your revision, log on to https://www.editorialmanager.com/pone/ and select the 'Submissions Needing Revision' folder to locate your manuscript file.

- A letter that responds to each point raised by the academic editor and reviewer(s). You should upload this letter as a separate file labeled 'Response to Reviewers'.
- A marked-up copy of your manuscript that highlights changes made to the original version. You should upload this as a separate file labeled 'Revised Manuscript with Track Changes'.
- An unmarked version of your revised paper without tracked changes. You should upload this as a separate file labeled 'Manuscript'.

If you would like to make changes to your financial disclosure, please include your updated statement in your cover letter. Guidelines for resubmitting your figure files are available below the reviewer comments at the end of this letter.

If applicable, we recommend that you deposit your laboratory protocols in protocols.io to enhance the reproducibility of your results. Protocols.io assigns your protocol its own identifier (DOI) so that it can be cited independently in the future. For instructions see: https://journals.plos.org/plosone/s/submission-guidelines#loc-laboratory-protocols. Additionally, PLOS ONE offers an option for publishing peer-reviewed Lab Protocol articles, which describe protocols hosted on protocols.io. Read more information on sharing protocols at https://plos.org/protocols?utm_medium=editorial-email&utm_source=authorletters&utm_campaign=protocols.

As the corresponding author, your ORCID iD is verified in the submission system and will appear in the published article. PLOS supports the use of ORCID, and we encourage all coauthors to register for an ORCID iD and use it as well. Please encourage your coauthors to verify their ORCID iD within the submission system before final acceptance, as unverified ORCID iDs will not appear in the published article. *Only*  the individual author can complete the verification step; PLOS staff *cannot*  verify ORCID iDs on behalf of authors.

We look forward to receiving your revised manuscript.

Kind regards,

Zhibin Zhao

Academic Editor

PLOS One

Journal Requirements:

When submitting your revision, we need you to address these additional requirements.

1.Please ensure that your manuscript meets PLOS ONE's style requirements, including those for file naming. The PLOS ONE style templates can be found at

https://journals.plos.org/plosone/s/file?id=wjVg/PLOSOne_formatting_sample_main_body.pdf and

https://journals.plos.org/plosone/s/file?id=ba62/PLOSOne_formatting_sample_title_authors_affiliations.pdf

2. Please note that PLOS One has specific guidelines on code sharing for submissions in which author-generated code underpins the findings in the manuscript. In these cases, we expect all author-generated code to be made available without restrictions upon publication of the work. Please review our guidelines at https://journals.plos.org/plosone/s/materials-and-software-sharing#loc-sharing-code and ensure that your code is shared in a way that follows best practice and facilitates reproducibility and reuse.

3. Thank you for stating the following financial disclosure:

“This paper is supported by the project 2025TSGCCZZB0911 supported by Key R&D Program of Shandong Province (Innovation Capability Enhancement Project for Technology based Small and Medium sized Enterprises).”

Please state what role the funders took in the study.  If the funders had no role, please state: "The funders had no role in study design, data collection and analysis, decision to publish, or preparation of the manuscript."

If this statement is not correct you must amend it as needed.

Please include this amended Role of Funder statement in your cover letter; we will change the online submission form on your behalf.

4. In the online submission form, you indicated that your data is available only on request from a third party. Please note that your Data Availability Statement is currently missing [the name of the third party contact or institution / contact details for the third party, such as an email address or a link to where data requests can be made]. Please update your statement with the missing information.

5. Please ensure that you refer to Figures 8 and 9 in your text as, if accepted, production will need this reference to link the reader to the figure.

6. We notice that your supplementary figures are uploaded with the file type 'Figure'. Please amend the file type to 'Supporting Information'. Please ensure that each Supporting Information file has a legend listed in the manuscript after the references list.

7. If the reviewer comments include a recommendation to cite specific previously published works, please review and evaluate these publications to determine whether they are relevant and should be cited. There is no requirement to cite these works unless the editor has indicated otherwise.

Additional Editor Comments:

The reviewer gave some useful comments, the author should revise the paper comprehensively before acceptance.

[Note: HTML markup is below. Please do not edit.]

Reviewer's Responses to Questions

**Comments to the Author**

1. Is the manuscript technically sound, and do the data support the conclusions?

Reviewer #1: Yes

Reviewer #2: Yes

**********

2. Has the statistical analysis been performed appropriately and rigorously?

Reviewer #1: Yes

Reviewer #2: Yes

**********

3. Have the authors made all data underlying the findings in their manuscript fully available?

The PLOS Data policy

Reviewer #1: No

Reviewer #2: Yes

**********

4. Is the manuscript presented in an intelligible fashion and written in standard English?

Reviewer #1: Yes

Reviewer #2: Yes

**********

Reviewer #1: This paper proposes several modules, including the guided three-channel input, a Multi-scale Omni-kernel Convolutions (MOM-Conv) backbone, and a Dual-Scale Wavelet Mamba (DSW-Mamba) structure. Experimental results demonstrate that these designs exhibit strong robustness in bearing fault diagnosis. Nevertheless, the overall method still has certain limitations that require further improvement.

(1) The author mentioned in Section 2.4 "Dual-Scale Wavelet Mamba Transform (DSW-Mamba)" that although the computational complexity of the Mamba architecture has been reduced to a linear level, when directly applied to noisy high-frequency vibration signals, it is prone to overfitting. However, no theoretical basis has been provided for its adaptability to low-frequency signals. It is suggested to provide supplementary explanations..

(2) It is suggested that a summary and explanation of the changes in the five different states be included in the visualization graph of the DSW-Mamba Block output results shown in Figure 3.

(3) The Mamba module is one of the important components in this article. However, the author does not provide a detailed explanation of the applicability and design purpose of the Mamba module in the text.It is suggested that the author add the principle diagram and related explanations of the Mamba Block within the DSW-Mamba module.

(4) In Table 7, the inference speed of this model is at the slowest level, but there is no explanation for this in the text.

(5) In Section 4.3 "Ablation Study of Key Components", Table 8 merely shows the impact of different individual modules on the overall performance of the model, without reflecting the performance of different combined modules on the overall model performance. This lacks sufficient persuasiveness.

(6) While the proposed wavelet-Mamba network demonstrates impressive robustness under noisy conditions, the manuscript could benefit from a deeper discussion on the interpretability of the learned features. In the field of safety-critical industrial applications, understanding why the model makes a certain diagnosis is as important as the accuracy itself. The authors are encouraged to discuss how their method aligns with recent developments in Explainable Artificial Intelligence (XAI) for fault diagnosis. For instance, recent studies have systematically reviewed XAI applications in this domain (e.g., Reliability Engineering & System Safety) or proposed novel interpretable architectures integrating wavelet theories like the Kolmogorov–Arnold-informed graph networks (e.g., IEEE Transactions on Systems, Man, and Cybernetics: Systems). Incorporating such discussions would significantly enhance the paper's contribution to the ongoing debate on black-box vs. interpretable models.

Reviewer #2: This manuscript proposes MDW-Mamba, a lightweight bearing fault diagnosis framework for rotating machinery under noisy and variable operating conditions. The method combines multi-scale omni-kernel convolution, and a dual-scale wavelet-Mamba module to improve robustness against broadband noise. Experiments on Ottawa, XJTU-SY, and JNU datasets show strong performance under low SNR conditions. However, the manuscript requires revision before publication.

1. Important implementation details are missing or vague, such as sampling frequency, segment length generation, overlap ratio, number of samples per class, train/validation/test split, batch size, learning rate, weight decay value, number of runs, random seeds.

2. The manuscript claims novelty through wavelet-guided Mamba and noise-feature decoupling, but the distinction from prior wavelet-Mamba or Mamba-based fault diagnosis methods needs to be sharpened.

3. Several figures appear low-resolution or cropped, especially the architecture and comparison plots.

4. The authors should clarify whether the same hyperparameters were used for all baseline models or tuned separately for each model.

5. The manuscript should explain why Haar wavelet/db1 was selected and whether other wavelet bases were tested.

6. References should be standardized and updated with more recent 2023–2026 studies, such as 10.1109/tii.2023.3345449, 10.1016/j.knosys.2022.110172.

**********

what does this mean?). If published, this will include your full peer review and any attached files.

If you choose “no”, your identity will remain anonymous but your review may still be made public.

**Do you want your identity to be public for this peer review?**  For information about this choice, including consent withdrawal, please see our Privacy Policy

Reviewer #1: No

Reviewer #2: **Yes:**  Chuancang Ding

**********

[NOTE: If reviewer comments were submitted as an attachment file, they will be attached to this email and accessible via the submission site. Please log into your account, locate the manuscript record, and check for the action link "View Attachments". If this link does not appear, there are no attachment files.]

To ensure your figures meet our technical requirements, please review our figure guidelines: https://journals.plos.org/plosone/s/figures

You may also use PLOS’s free figure tool, NAAS, to help you prepare publication quality figures: https://journals.plos.org/plosone/s/figures#loc-tools-for-figure-preparation.

NAAS will assess whether your figures meet our technical requirements by comparing each figure against our figure specifications.

# Author comment: Author response to Decision Letter 1

**DOI:** 10.1371/journal.pone.0358759.r002

26 Jun 2026

Response to Reviewers

Manuscript ID: PONE-D-26-00901

Title: Robust bearing fault diagnosis for rotating machinery under noisy and variable operating conditions using a wavelet-Mamba network

Journal: PLOS ONE

Dear Editor and Reviewers,

We sincerely thank the Academic Editor, Dr. Zhibin Zhao, and both reviewers for the thorough and constructive evaluation. We have carefully addressed every point raised. Below is our point-by-point response. All changes in the revised manuscript are marked with track changes.

============================================================

Part I: Responses to Journal Requirements

============================================================

J1. PLOS ONE Style Requirements

Comment: Please ensure that your manuscript meets PLOS ONE's style requirements, including those for file naming.

Response: Thank you for this reminder. The revised manuscript has been reformatted according to the PLOS ONE style templates. File naming follows the journal's conventions.

J2. Code Sharing

Comment: We expect all author-generated code to be made available without restrictions upon publication.

Response: Thank you for this important requirement. The complete source code for the proposed MDW-Mamba framework, including model architecture, training pipeline, evaluation scripts, and data preprocessing utilities for all three benchmark datasets (Ottawa, XJTU-SY, JNU), has been made publicly available on GitHub: https://github.com/Newdawn01/mdw_mamba. The repository includes a detailed README with setup instructions, usage examples, and hyperparameter configurations.

J3. Role of Funder Statement

Comment: Please state what role the funders took in the study.

Response: Thank you for this clarification request. The funders had no role in study design, data collection and analysis, decision to publish, or preparation of the manuscript. This amended statement is included in the cover letter.

J4. Data Availability Statement

Comment: The Data Availability Statement is currently missing the name of the third party contact or institution / contact details.

Response: Thank you for pointing out this omission. The Data Availability Statement has been updated with full contact information. All three datasets are publicly available: the Ottawa dataset from the University of Ottawa (DOI: 10.1016/j.dib.2018.11.019); the XJTU-SY dataset from Xi'an Jiaotong University (DOI: 10.3901/JME.2019.16.001); and the JNU dataset from Jiangnan University (DOI: 10.1016/j.isatra.2020.08.010).

J5. Figures 8 and 9 References

Comment: Please ensure that you refer to Figures 8 and 9 in your text.

Response: Thank you for this technical reminder. Figures 8 and 9 are properly referenced in the text (Section 4.2.1). All figure cross-references have been verified.

J6. Supplementary Figure File Type

Comment: Please amend the file type of supplementary figures to 'Supporting Information'.

Response: Thank you for this reminder. We have carefully re-examined our submission. The 12 uploaded image files correspond to Figures 1-12 of the main manuscript, all cited and discussed in the manuscript body. No supplementary figures are included in this submission. All figures are therefore correctly categorized as 'Figure' type, consistent with PLOS ONE guidelines.

J7. Citation Recommendations

Comment: If the reviewer comments include a recommendation to cite specific previously published works, please review and evaluate these publications.

Response: Thank you for this guidance. We have carefully reviewed the recommended publications and found them directly relevant. The following works are now cited in the revised manuscript:

- Shi et al. (2024), IEEE TII, DOI: 10.1109/TII.2023.3345449

- Shi et al. (2023), Knowledge-Based Systems, DOI: 10.1016/j.knosys.2022.110172

Both references have been verified against their official metadata.

============================================================

Part II: Response to Academic Editor

============================================================

Editor Comment: The reviewer gave some useful comments, the author should revise the paper comprehensively before acceptance.

Response: We thank the Editor for this guidance. We have comprehensively revised the manuscript, addressing every comment from both reviewers. Key additions include: (1) a theoretical justification for Mamba's low-frequency modeling capability (Section 2.4); (2) detailed implementation specifications (Section 4.1); (3) combined ablation experiments (Section 4.3, new Table 9); (4) an interpretability discussion connecting to XAI literature (Section 5.3); and (5) a wavelet basis selection study (Section 5.4, new Table 11).

============================================================

Part III: Response to Reviewer #1

============================================================

We sincerely thank Reviewer #1 for the rigorous and expert evaluation. We have carefully addressed each comment below.

R1-1. Theoretical Basis for Mamba's Low-Frequency Adaptability

Comment: The author mentioned in Section 2.4 that although the computational complexity of the Mamba architecture has been reduced to a linear level, when directly applied to noisy high-frequency vibration signals, it is prone to overfitting. However, no theoretical basis has been provided for its adaptability to low-frequency signals. It is suggested to provide supplementary explanations.

Response: Thank you for identifying this theoretical gap. We have added a detailed explanation in Section 2.4. The justification draws from the state-space model (SSM) formulation: the continuous-time dynamics matrix A governs how past information propagates or attenuates, functioning as an implicit low-pass filter. The Zero-Order Hold discretization with timescale parameter Delta introduces a Nyquist-type constraint --- frequencies exceeding 1/(2*Delta) are inherently aliased. When raw high-frequency signals with broadband noise are directly fed into Mamba, stochastic noise fluctuations force the state transition matrices to fit spurious high-frequency patterns, causing overfitting. Conversely, the low-frequency approximation coefficients X_L produced by DWT possess a compact spectral distribution matching the SSM's implicit bandwidth constraints, enabling stable long-range dependency modeling.

R1-2. Summary of Five Health States in Figure 3 t-SNE Visualization

Comment: It is suggested that a summary and explanation of the changes in the five different states be included in the visualization graph of the DSW-Mamba Block output results shown in Figure 3.

Response: Thank you for this practical suggestion. Figure 3 has been updated with explicit health state labels (H, O, I, B, C) and consistent color coding (blue, orange, green, red, purple) in the t-SNE panel. The figure caption identifies all five states and notes that the distinct clustering demonstrates the module's capability to disentangle fault-sensitive features. The accompanying text in Section 2.4 confirms that the five health states are mapped into compact and clearly separated clusters, indicating effective noise-feature decoupling.

R1-3. Mamba Block Principle Diagram and Explanation

Comment: The Mamba module is one of the important components in this article. However, the author does not provide a detailed explanation of the applicability and design purpose of the Mamba module in the text. It is suggested that the author add the principle diagram and related explanations of the Mamba Block within the DSW-Mamba module.

Response: Thank you for this recommendation. Figure 3 has been enhanced with an inset diagram detailing the internal structure of the Mamba block. We have added a comprehensive description in Section 2.4 elaborating the five-stage architecture: (1) Linear projection for feature expansion; (2) 1D causal convolution for local context encoding; (3) Selective state-space scan with input-dependent parameterization; (4) Gated output via element-wise multiplication with a SiLU-activated branch; and (5) Residual connection with layer normalization. The design purpose --- capturing long-range dependencies with O(L) complexity while selectively suppressing noise in the fault-specific low-frequency subband --- is explicitly discussed.

R1-4. Inference Speed Explanation (Table 7)

Comment: In Table 7, the inference speed of this model is at the slowest level, but there is no explanation for this in the text.

Response: Thank you for this pertinent observation. We have added a quantitative analysis in Section 4.2.2. The per-sample latency of 1.285 ms is attributed to the DWT/IDWT operations and the serial selective scan mechanism. This latency remains well below the real-time constraint of 10 ms for industrial monitoring, corresponding to approximately 780 samples per second throughput. Given the approximately 20% accuracy improvement over faster models under low SNR, this moderate trade-off is acceptable. We also discuss potential optimization strategies including kernel fusion and operator pruning.

R1-5. Combined Module Ablation Study (Table 8/9)

Comment: Table 8 merely shows the impact of different individual modules on the overall performance of the model, without reflecting the performance of different combined modules on the overall model performance. This lacks sufficient persuasiveness.

Response: Thank you for this critical suggestion. We have conducted combined ablation experiments, presented in a new Table 9 (Section 4.3). Among the 26 possible multi-module combinations, four representative configurations were selected based on three explicit constraints: (1) Orthogonal processing stages --- each pair targets a distinct functional interaction axis (input preprocessing, time-domain feature extraction, frequency-domain refinement, training regularization); (2) Full module coverage --- all five key modules (3-Channel, MOMConv, DWT, Mamba, Aug) appear in at least one ablated combination; (3) Non-redundancy --- configurations that would reduce to near-equivalent residual architectures are excluded. Results across all three datasets confirm that the five modules contribute complementary and non-redundant features, with simultaneous removal causing greater degradation than any single-module removal alone.

R1-6. Interpretability and Connection to XAI

Comment: The manuscript could benefit from a deeper discussion on the interpretability of the learned features. The authors are encouraged to discuss how their method aligns with recent developments in Explainable Artificial Intelligence (XAI) for fault diagnosis.

Response: Thank you for this forward-looking suggestion. We have added a new subsection "Interpretability Considerations and Connection to XAI" (Section 5.3). We discuss how MDW-Mamba embodies ante-hoc interpretability: the DSW-Mamba module's DWT-based decomposition provides physically meaningful frequency subband separation; the three-channel input construction enables channel-wise ablation to quantify each statistical prior's contribution; and the frequency-domain decoupling strategy aligns with the XAFD taxonomy proposed by Li et al. (Reliability Engineering & System Safety, DOI: 10.1016/j.ress.2025.111935). We also connect our work to the Kolmogorov-Arnold-informed graph wavelet networks recommended by the reviewer (Li et al., IEEE TSMC, DOI: 10.1109/TSMC.2025.3647102) and discuss learnable wavelet parameters as a future direction.

============================================================

Part IV: Response to Reviewer #2

============================================================

We sincerely thank Reviewer #2 for the meticulous and methodologically rigorous evaluation. We have carefully addressed each comment below.

R2-1. Missing Implementation Details

Comment: Important implementation details are missing or vague, such as sampling frequency, segment length generation, overlap ratio, number of samples per class, train/validation/test split, batch size, learning rate, weight decay value, number of runs, random seeds.

Response: Thank you for this thorough checklist. We have added a comprehensive "Experimental Setup and Implementation Details" subsection (Section 4.1) specifying every item raised:

- Software framework: PyTorch 2.6

- Sampling frequencies: Ottawa 200 kHz, XJTU-SY 25.6 kHz, JNU 50 kHz

- Segment length: L = 1024, no overlap (stride = L)

- Samples per class: approximately 500-2,000 depending on dataset and condition

- Data split: 80% training / 20% test, stratified random split, seed = 42

- Noise injection: AWGN applied to test set only; training on clean data

- Training: Mixed-condition training across all operating conditions

- Optimizer: AdamW, lr = 3e-4, weight decay = 1e-4, beta = (0.9, 0.999)

- Learning rate schedule: Cosine annealing, minimum lr = 1e-6

- Epochs: 100, batch size = 32

- Number of runs: 10 independent repetitions with different random seeds

- Reported metrics: Mean accuracy +/- standard deviation across 10 runs

R2-2. Distinction from Prior Wavelet-Mamba Methods

Comment: The manuscript claims novelty through wavelet-guided Mamba and noise-feature decoupling, but the distinction from prior wavelet-Mamba or Mamba-based fault diagnosis methods needs to be sharpened.

Response: Thank you for this incisive comment. We have added an explicit comparative discussion in the Introduction, identifying two fundamental differences:

(1) Noise decoupling vs. feature enrichment: Prior works (e.g., Yang et al., 2025; Lai et al., 2025) employ wavelets primarily for multi-resolution feature enrichment or multi-channel fusion, without addressing the physical separation of fault features from broadband noise. Our DSW-Mamba module implements a frequency-domain "divide-and-conquer" strategy where DWT orthogonally decomposes the signal, and Mamba selectively models only the low-frequency, fault-dominant components.

(2) Frequency-selective scanning: Unlike existing Mamba-based methods that process full-band signals, MDW-Mamba's selective state-space scan operates exclusively on compact, DWT-decomposed low-frequency coefficients. This fundamentally prevents overfitting to high-frequency noise patterns. At SNR = -6 dB, MDW-Mamba achieves approximately 87% accuracy, surpassing competing models by approximately 20 percentage points.

This distinction is reinforced in the updated Contribution (2) statement.

R2-3. Low-Resolution or Cropped Figures

Comment: Several figures appear low-resolution or cropped, especially the architecture and comparison plots.

Response: Thank you for bringing this to our attention. All figures have been re-processed through the PLOS ONE NAAS (New Automated Article Submission) tool, which automatically standardizes image dimensions to journal specifications and verifies resolution compliance. All 12 figures now pass the NAAS technical compliance check with "Image complies with specification" status. The architecture diagram (Figure 1), comparison plots (Figure 10), and all other figures have been verified at 300 DPI or higher.

R2-4. Baseline Model Hyperparameter Configuration

Comment: The authors should clarify whether the same hyperparameters were used for all baseline models or tuned separately for each model.

Response: Thank you for this important question. We have added an explicit statement in Section 4.1: "All baseline models use the same data splits, preprocessing pipeline, optimizer settings, and evaluation protocol as MDW-Mamba. Architectural hyperparameters for each baseline model follow the specifications reported in their original publications. No dataset-specific or model-specific hyperparameter tuning was performed beyond the published configurations, ensuring a fair and reproducible comparison."

R2-5. Wavelet Basis Selection

Comment: The manuscript should explain why Haar wavelet/db1 was selected and whether other wavelet bases were tested.

Response: Thank you for this methodological suggestion. We have added a new subsection "Wavelet Basis Selection" (Section 5.4) with a comparative study across three Daubechies wavelet bases (db1/Haar, db2, db4). The results (Table 11) demonstrate that the Haar wavelet achieves competitive or superior performance to higher-order wavelets across all three datasets and SNR levels, while offering the lowest computational complexity (support width = 2, single vanishing moment). This makes db1 the optimal choice for resource-constrained edge deployment. All results are presented as mean +/- standard deviation across ten experimental runs.

R2-6. References Updated with Recent Studies

Comment: References should be standardized and updated with more recent 2023-2026 studies, such as 10.1109/TII.2023.3345449, 10.1016/j.knosys.2022.110172.

Response: Thank you for these literature recommendations. We have updated the reference list with the recommended works and additional recent studies (2023-2026):

- Shi et al. (2024), IEEE TII, DOI: 10.1109/TII.2023.3345449

- Shi et al. (2023), Knowledge-Based Systems, DOI: 10.1016/j.knosys.2022.110172

- Li et al. (2026), Reliability Engineering & System Safety, DOI: 10.1016/j.ress.2025.111935

- Li et al. (2026), IEEE TSMC, DOI: 10.1109/TSMC.2025.3647102

All references have been verified against their official metadata and formatted according to Vancouver style.

We hope the revised manuscript now satisfactorily addresses all concerns. We remain at your disposal for any further questions.

Sincerely,

Dayou Cui, Zhaoyan Xie, Zhixue Wang, Xiaowei Li, Xiangxuan Meng

Attachment Submitted filename: Response_to_Reviewers.pdf

# Peer review: Decision Letter 1

**DOI:** 10.1371/journal.pone.0358759.r003

**Authors:** Zhibin Zhao

7 Sep 2026

Robust bearing fault diagnosis for rotating machinery under noisy and variable operating conditions using a wavelet-Mamba network

PONE-D-26-00901R1

Dear Dr. Xie,

We’re pleased to inform you that your manuscript has been judged scientifically suitable for publication and will be formally accepted for publication once it meets all outstanding technical requirements.

Within one week, you’ll receive an e-mail detailing the required amendments. When these have been addressed, you’ll receive a formal acceptance letter and your manuscript will be scheduled for publication.

An invoice will be generated when your article is formally accepted. Please note, if your institution has a publishing partnership with PLOS and your article meets the relevant criteria, all or part of your publication costs will be covered. Please make sure your user information is up-to-date by logging into Editorial Manager at Editorial Manager® and clicking the ‘Update My Information' link at the top of the page. For questions related to billing, please contact billing support.

If your institution or institutions have a press office, please notify them about your upcoming paper to help maximize its impact. If they’ll be preparing press materials, please inform our press team as soon as possible -- no later than 48 hours after receiving the formal acceptance. Your manuscript will remain under strict press embargo until 2 pm Eastern Time on the date of publication. For more information, please contact onepress@plos.org.

Kind regards,

Zhibin Zhao

Academic Editor

PLOS One

Additional Editor Comments (optional):

I have reviewed comments, the authors have well answered all the issues.

Reviewers' comments:

Reviewer's Responses to Questions

**Comments to the Author**

Reviewer #1: All comments have been addressed

**********

2. Is the manuscript technically sound, and do the data support the conclusions?

Reviewer #1: Yes

**********

3. Has the statistical analysis been performed appropriately and rigorously?

Reviewer #1: Yes

**********

4. Have the authors made all data underlying the findings in their manuscript fully available?

The PLOS Data policy

Reviewer #1: Yes

**********

5. Is the manuscript presented in an intelligible fashion and written in standard English?

Reviewer #1: Yes

**********

Reviewer #1: This paper proposes a robust and lightweight bearing fault diagnosis framework. I have no further question, this paper can be accepted.

**********

what does this mean?). If published, this will include your full peer review and any attached files.

If you choose “no”, your identity will remain anonymous but your review may still be made public.

**Do you want your identity to be public for this peer review?**  For information about this choice, including consent withdrawal, please see our Privacy Policy

Reviewer #1: No

**********

# Editor report: Acceptance letter

**DOI:** 10.1371/journal.pone.0358759.r004

**Authors:** Zhibin Zhao

PONE-D-26-00901R1

PLOS One

Dear Dr. Xie,

I'm pleased to inform you that your manuscript has been deemed suitable for publication in PLOS One. Congratulations! Your manuscript is now being handed over to our production team.

At this stage, our production department will prepare your paper for publication. This includes ensuring the following:

* All references, tables, and figures are properly cited

* All relevant supporting information is included in the manuscript submission,

* There are no issues that prevent the paper from being properly typeset

You will receive further instructions from the production team, including instructions on how to review your proof when it is ready. Please keep in mind that we are working through a large volume of accepted articles, so please give us a few days to review your paper and let you know the next and final steps.

Lastly, if your institution or institutions have a press office, please let them know about your upcoming paper now to help maximize its impact. If they'll be preparing press materials, please inform our press team within the next 48 hours. Your manuscript will remain under strict press embargo until 2 pm Eastern Time on the date of publication. For more information, please contact onepress@plos.org.

You will receive an invoice from PLOS for your publication fee after your manuscript has reached the completed accept phase. If you receive an email requesting payment before acceptance or for any other service, this may be a phishing scheme. Learn how to identify phishing emails and protect your accounts at https://explore.plos.org/phishing.

If we can help with anything else, please email us at customercare@plos.org.

Thank you for submitting your work to PLOS One and supporting open access.

Kind regards,

PLOS One Editorial Office Staff

on behalf of

Dr. Zhibin Zhao

Academic Editor

PLOS One
